# Autophagy in ocular diseases: from mechanisms to therapeutic potential

**DOI:** 10.3389/fcell.2026.1727005

**Published:** 2026-02-24

**Authors:** Qi Zhao, Yumeng Lin, Zhongyu Han, Yiwei Tian, Yaqi Yang, Qiaoyin Gou, Yuan Ju, Duoduo Xu, Lijuan Wei

**Affiliations:** 1 College of Chinese Medicine, Changchun University of Chinese Medicine, Changchun, China; 2 Department of Nanjing Tongren Eye Center, Nanjing Tongren Hospital, School of Medicine, Southeast University, Nanjing, China; 3 Institute of Nephrology, Zhongda Hospital, Southeast University, Nanjing, China; 4 Ophthalmology Department, Affiliated Hospital of Changchun University of Chinese Medicine, Changchun, China; 5 Chengdu Shuangliu District Huangjia Community Health Service Center, Chengdu, China; 6 School of Pharmacy, Changchun University of Chinese Medicine, Jilin, China

**Keywords:** autophagy, corneal disease, glaucoma, mitophagy, retina

## Abstract

Autophagy represents a fundamental and evolutionarily preserved mechanism of degradation and metabolism in eukaryotic cells. This process is triggered by a variety of stressors, including nutrient deprivation, energy deficits, protein misfolding, low oxygen levels, and pathogen infections by pathogens. Autophagy plays a vital role in maintaining cellular equilibrium. The process of vision is notably complex, making the eye one of the most metabolically active tissues in the human body. The proper function of the eye relies on the preservation of metabolic homeostasis and the structural integrity of organelles within various types of cells, including those found in the cornea, lens, retina, and optic nerve. As a result, any disruption in autophagy is closely linked to numerous ocular conditions. This review meticulously examines and elucidates the role of autophagy in ophthalmic diseases and explores its involvement in disease progression and treatment strategies, with the aim of presenting potential therapeutic approaches and a foundational framework for future research into the management of ophthalmic disorders.

## 1 Introduction

Autophagy is a fundamental homeostatic process that facilitates the degradation of proteins, lipids, and impaired organelles via the autophagosomal–lysosomal pathway, thereby enabling the recycling of intracellular constituents (Dikic and Elazar, 2018). Autophagy, however, plays a paradoxical role in cellular fate. While it is required for cellular homeostasis, protecting cells by eliminating damaged organelles and protein aggregates, it can also be detrimental. The dysregulation of autophagy, whether it is insufficient or overactivated, disrupts intracellular metabolism and can induce programmed cell death.

Consequently, the maintenance of cellular homeostasis and health is crucially dependent on the tight regulation of autophagic activity (Liu S. et al., 2023). Recent investigations have revealed a significant association between autophagy and various conditions including cancer, immune disorders, and neurodegenerative diseases, indicating that autophagy holds substantial promise for therapeutic interventions.

Ocular tissues encounter numerous environmental challenges and stresses, and autophagy is crucial for preserving ocular homeostasis (Jiménez-Loygorri et al., 2023). Functioning as the primary barrier against external hazards, the cornea employs autophagy to eliminate misfolded proteins and damaged organelles. This mechanism is crucial for preserving the integrity of the tight junctions that connect corneal epithelial cells, thus enhancing the cornea’s ability to withstand the invasion of external physical agents, chemical substances, and pathogenic microorganisms (Vallabh et al., 2017). Retinal photoreceptors are continuously exposed to light and are prone to oxidative stress damage, requiring autophagy to maintain photoelectric conversion efficiency by removing photo-oxidatively damaged mitochondria and endoplasmic reticulum. At the same time, the retinal pigment epithelial (RPE) cell degrade shed photoreceptor outer segment disc membranes through autophagy to prevent the abnormal accumulation of metabolites such as lipofuscin (Krohne et al., 2010). In addition, lens fiber cells rely on autophagy to remove organelle remnants and maintain transparency, and retinal ganglion cells acquire energy through autophagy to ensure long-distance transport of substances, such as neurotransmitter precursors, along axons (Brennan et al., 2023).

Consequently, impaired of autophagy is significantly associated with the pathological progression of numerous eye disorders, including degeneration of RPE cells in age-related macular degeneration (AMD), apoptosis of retinal ganglion cells in glaucoma, and dysfunction of the vascular endothelium in diabetic retinopathy. These findings underscore the critical importance of autophagy in the pathogenesis of ocular diseases.

In this review, we systematically elucidate the molecular regulatory mechanism of autophagy in ophthalmic diseases, analyse its pathological association with common eye diseases, summarize the latest research progress in of autophagy-targeted therapy, provide new ideas and theoretical basis for ophthalmic disease mechanism research and clinical intervention, and provide new ideas and a theoretical basis for ophthalmic disease mechanisms research and clinical intervention.

## 2 Autophagy

**FIGURE 1:**
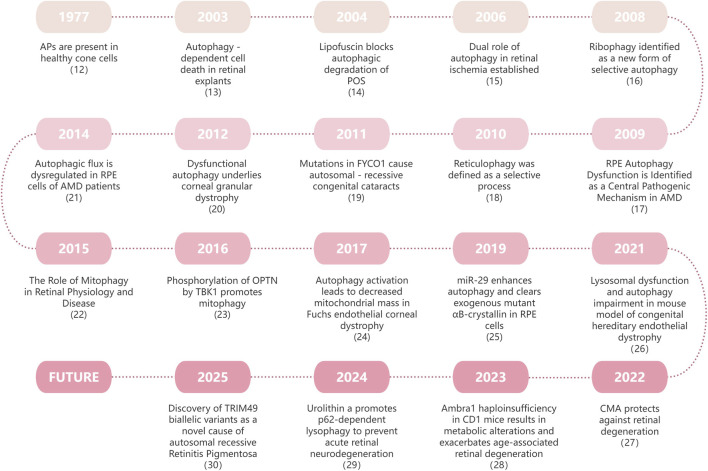
Timeline of autophagy and eye research. This timeline systematically outlines the key developments in autophagy research within ophthalmology. It spans foundational discoveries, from the initial observation of cytoplasm-derived membranous structures by Clark in 1957, to groundbreaking breakthroughs in 2025, such as the identification of biallelic TRIM49 variants as a novel cause of autosomal recessive retinitis pigmentosa.

Autophagy represents a prominent degradation mechanism that occurs across eukaryotic organisms, and is responsible for the breakdown of aged, damaged, or surplus organelles and proteins within cells (Dikic and Elazar, 2018). This process occurs via lysosomal activity and yields various metabolites, including amino acids and free fatty acids, thereby facilitating energy recycling. On the one hand, it supports cellular metabolism and sustains survival under in conditions of starvation and stress, while on the other hand, it facilitates the removal of damaged proteins and organelles, thereby preserving both the quality and quantity of these cellular components (Ghavami et al., 2014). Under nutrient-rich conditions, cells perform only basal autophagy to control the quality of the intracellular environment, provide recycling mechanisms for cytoplasmic components to tissues, and remove damaged or unnecessary organelles. In the face of diverse unfavourable conditions, such as hypoxia, intracellular infections, and chemical stressors, the rate of autophagy within cells is markedly imbalanced compared with that under basal conditions. This increase in autophagy serves to sustain the levels of amino acids within the cytoplasm, thereby facilitating the efficient continuation of protein *de novo* protein synthesis, energy production, and gluconeogenesis. Autophagy provides cells with the energy and conditions required for survival and is a self-protective mechanism for cells within an organism (Hao et al., 2022). This central role of autophagy in maintaining cellular homeostasis has led to significant advances in understanding its mechanisms in eye diseases, as outlined in the timeline below (Figure 1) (Liton et al., 2023; Guimaraes et al., 2003; Bergmann et al., 2004; Baehrecke, 2005; Munemasa et al., 2008; Kaarniranta, 2009; Chen et al., 2011; Ferrier et al., 2015; Mitter et al., 2014; Thomas et al., 2015; Heo et al., 2015; Benischke et al., 2017; Cai et al., 2019; Shyam et al., 2021; Seki et al., 2021; Jiménez-Loygorri et al., 2024; Yi et al., 2025; Knöferle et al., 2010; Ramírez-Pardo et al., 2023).

### 2.1 Types of autophagy

Autophagy is classified into three distinct types based on the mechanism of substrate delivery into lysosomes. Macroautophagy involves the encapsulation of damaged cytoplasmic organelles within double-membraned autophagosomes. These autophagosomes subsequently fuse with lysosomes to form autolysosomes where the enclosed materials are degraded. In contrast microautophagy occurs through the direct invagination of the lysosomal membrane which engulfs substrates for degradation without the involvement of autophagosomes. Moreover, chaperone-mediated autophagy represents a selective process in which substrate proteins are recognized by chaperone complexes transported to specific receptors on the lysosomal membrane and subsequently internalized for lysosomal degradation (Nikoletopoulou et al., 2015).

Autophagy can be categorized into two distinct types based on its substrate selectivity: non-selective autophagy and selective autophagy. These two forms exhibit significant differences across various dimensions. Non-selective autophagy is triggered by a lack of nutrients and serves the purpose of randomly enveloping and breaking down various cytoplasmic elements, such as organelles and large molecular assemblies. This process is crucial for sustaining cellular viability while awaiting the availability of fresh nutrient supplies. This mechanism is vital for maintaining cellular viability during periods of nutrient scarcity. In non-selective autophagy, which does not require specific substrate recognition, the detection of nutrient deprivation by the cell triggers autophagic activation and leads to the formation of a specialized membrane structure known as the phagophore (Suzuki and Ohsumi, 2007). Selective autophagy subtypes and their associations with ocular diseases are summarized in Table 1. Many studies have shown that various organelles such as the endoplasmic reticulum, mitochondria, and cell membranes are involved in this process. Non-selective autophagy contains macroautophagy and microautophagy (Hamasaki et al., 2013).

**TABLE 1:** Selective autophagy and ocular disease.

Type	Concept	Ocular disease	Tissue targets	Mechanism	References
Mitophagy	Failure to clear dysfunctional mitochondria leads to ROS burst, activation of apoptotic signaling, and may trigger secondary cell death pathways. Its dysfunction is central to neurodegeneration and glandular function decline	AMD	RPE	PINK1 Parkin blockade activates ROS and inflammation methylation	Kaarniranta et al. (2020)
		Glaucoma	RGCs	Linked to OPTN mutation	D’Amico et al. (2024)
		DR	Photoreceptor cells	Involves PINK1/Parkin pathway	Li et al. (2025)
		Cataract	Lens epithelial cells	PINK1-mediated mitophagy provides cytoprotection	Liu X. et al. (2025)
		DED	Lacrimalgland cells	Mitophagy is attenuated in the lacrimal gland	Zhao et al. (2024)
		FECD	CEnCs	Mitophagy overdrive exhausts CEnCs and piles guttae	Kumar and Jurkunas (2021)
Lysophagy	Autophagosome lysosome fusion blocked substrates undegraded toxic proteins lipofuscin damaged organelles accumulate oxidative stress and inflammation amplified	AMD	RPE	The AKT2-SYTL1-TRIM16-SNAP23 pathway drives aberrant secretory autophagy in RPE.	Ghosh et al. (2024)
		Glaucoma	TM	ALP clears damaged organelles/proteins in TM cells to tune TGF-β and ECM remodeling	Shim and Liton (2022)
​		DED	HCEC	HMGB1 promotes disrupts lysosomal homeostasis to drive dry-eye lysosomal cell	Hu et al. (2025)
​	​	​	​	Modulated by exosomes (WNT/β-catenin, Nrf2)	​
ER-phagy	Inability to clear misfolded proteins and damaged ER fragments triggers sustained UPR and proteotoxic stress, ultimately leading to cell dysfunction, inflammation, and apoptosis	AMD	RPE	ISR inhibition blocks ATF4	Khater et al. (2022)
​		Glaucoma	TM cells	Modulated by exosomes (WNT/β-catenin, Nrf2)	Stothert et al. (2016)
​		DR	Retinal vasculature	TUDCA inhibits Grp78	Lenin et al. (2023)
​		Cataract	Lens epithelium	4-PBA inhibits ER stress	Yasuda et al. (2021)
Lipophagy	Impaired degradation of lipid droplets leads to abnormal lipid metabolism, lipofuscin deposition, oxidative damage, and chronic inflammation. Its dysfunction is closely linked to aging and metabolic diseases	AMD	RPE	Upregulation of LCN2 impairs lipophagy, leading to defective lipid clearance, ferroptosis	Gupta et al. (2023)
​		DED	CEnCs	Atg5-mediated lipophagy induces ferroptosis in CEnCs	Zuo et al. (2024)
Aggrephagy	Failure to efficiently clear toxic protein aggregates leads to proteotoxic accumulation, directly inducing cell degeneration and death	Glaucoma	Retinal neurons	OPTN targets aggregates	Ying and Yue (2016)
​		Cataract	Lens epithelium	RNF114-UPS clears A-crystallin	Yang H. et al. (2024)

RGCs, Retinal Ganglion Cells; TM cells, Trabecular Meshwork cells; RPE, Retinal Pigment Epithelium; DR, Diabetic Retinopathy; OPTN, Optineurin; PINK1, PTEN Induced Kinase; Nrf2, Nuclear factor erythroid 2–related factor; Grp78, Glucose-regulated protein; ATF4, Activating Transcription Factor; TFEB, Transcription Factor EB; RNF114, Ring Finger Protein 114; UPS, Ubiquitin-Proteasome System; DED, Dry Eye Disease; ISR, Integrated Stress Response; AMD, Age-related Macular Degeneration; TUDCA, Tauroursodeoxycholic Acid; 4-PBA, 4-Phenylbutyric Acid; HCEC, Human Corneal Epithelial Cell.

Selective autophagy refers to the targeted identification and elimination of particular substances that are often detrimental to cellular health, including damaged organelles, protein aggregates, and invading pathogens. This process plays a crucial role in maintaining cellular quality control. Selective autophagy can be further categorized into several types, including mitophagy, lysosome-associated autophagy, aggregate autophagy, foreign body autophagy, endoplasmic reticulum-autophagy, and peroxisome autophagy (Vargas et al., 2023). Among them, macroautophagy is the main degradation pathway of proteins in the cytosol and is also commonly known as autophagy in general, and the autophagy mentioned below refers to macroautophagy.

### 2.2 Autophagic process

Vesicles of autophagosomes are formed not by membrane budding but from scratch. Autophagy includes the initiation, formation and extension of sequestration membranes, autophagosome formation and maturation, the formation of the autophagy–lysosome system, and the degradation of substrates, and this process is regulated by a variety of factors and signalling pathways (Yu et al., 2017). Under normal physiological conditions, overall autophagic activity remains at a relatively low basal level, yet it displays significant cell-type specificity. Specialized postmitotic cells in the retina-such as retinal ganglion cells (RGCs), retinal pigment epithelial (RPE), and photoreceptors, require constitutively increased basal autophagy because of their elevated metabolic demands, persistent oxidative stress, and active cellular turnover (Yao et al., 2016). Thus, understanding autophagy, particularly in the context of retinal physiology and diseases such as age-related macular degeneration, necessitates consideration of such cell-type-specific.

Cellular stress initiates the formation of an open, double-membrane phagophore in the cytoplasm. Through extension and enclosure of cytoplasmic materials such as protein aggregates and dysfunctional organelles, this precursor matures into a closed autophagosome bounded by a bilayer membrane. These autophagosomes are subsequently delivered to lysosomes through microtubule-dependent transport (Ye and Zheng, 2021).

*In vitro* studies have demonstrated that autophagic membranes engage in contact and fusion with lysosomal membranes. These autophagic membranes, along with their enclosed materials, subsequently translocate into the lysosomal lumen, leading to the formation of autolysosomes. These structures subsequently the undergo acidification, which triggers the activation of acidic proteolytic enzymes within the lysosomes. This enzymatic activity facilitates the degradation of aged or impaired proteins and organelles, resulting in the release of amino acids, free fatty acids, and other metabolites that are available for cellular metabolic processes. This complete process is called autophagic flux; the blockade of any step impairs autophagic flux (Sarkar, 2013).

### 2.3 Regulation of autophagy

Proteins that are encoded by genes associated with autophagy, known as autophagy-related targeted genes (ATG), are crucial for the autophagic mechanism. More than 40 ATG genes have been identified, including core ATG genes (*ATG1*–*ATG18*). Their encoded proteins are evolutionarily conserved, maintaining sequence and functional consistency from yeast to mammals and humans. This high degree of conservation provides foundational support for utilizing model organisms in autophagy research, thereby facilitating mechanistic insights into human diseases and the development of therapeutic strategies (Vargas et al., 2023).

The initiation of autophagy is primarily regulated by the ULK1 complex (ULK1, FIP200, ATG13, and ATG101). Another category features ATG9, distinguished as the sole ATG protein possessing multiple transmembrane domains, which is involved in membrane sourcing and its trafficking is regulated by ULK1/AMPK-mediated phosphorylation (Ren et al., 2022).

The PI3KC3 complex constitutes a further group, which primarily includes Beclin-1, VPS34, p150, NRBF2, and ATG14L, or alternatively, Beclin-1, VPS34, UVRAG, and p150 (Ma et al., 2017). Crucially, Beclin-1 functions as a critical signaling hub that integrates diverse upstream regulatory inputs beyond mere structural scaffolding. Its activity is dynamically controlled by multiple interactors, including inhibitory proteins such as BCL-2 and BCL-XL (which bind Beclin-1 under nutrient-rich conditions to suppress autophagy) and activating regulators including AMBRA1, UVRAG, and HMGB1. Furthermore, Beclin-1-dependent VPS34 activity is modulated by extensive post-translational modifications, including phosphorylation by ULK1, DAPK1, and mTOR, as well as ubiquitination by TRAF6 or NEDD4, enabling precise spatiotemporal control of autophagy initiation in response to cellular stress signals (Proikas-Cezanne et al., 2015; Valverde et al., 2019).

Lastly, the ubiquitin-like protein conjugation system encompasses the ATG5-ATG12-ATG16L1 complex along with the ATG8/LC3/GABARAP family, which are essential for membrane elongation and closure (Komatsu et al., 2005).

Mammalian cells precisely modulate the initiation of autophagy through key signaling kinases in response to environmental fluctuations, with the core regulation residing in the deeply coupled and functionally antagonistic system of AMP-activated protein kinase (AMPK) and the mechanistic target of rapamycin complex 1 (mTORC1) (Xue et al., 2024). AMPK acts as the cellular energy sensor, activated under energy stress (e.g., increased AMP/ATP ratio) by kinases such as LKB1, thereby initiating catabolic processes. In contrast, mTORC1 serves as the master regulator of anabolism, activated when nutrients and energy are abundant, promoting biosynthesis and suppressing autophagy. Together, they form the core regulatory module for cellular growth/survival decisions.

Their coupling is manifested through multi-layered, bidirectional, and dynamic regulation. Under nutrient-rich conditions, active mTORC1 potently inhibits autophagy initiation by phosphorylating components of the ULK1 complex, namely ULK1 and ATG13 (Hosokawa et al., 2009). Conversely, during nutrient deprivation or energy stress, AMPK is activated and concomitantly suppresses mTORC1 through a dual mechanism: first, by directly phosphorylating RAPTOR, a critical component of mTORC1; and second, by activating the TSC2 complex, thereby persistently inhibiting Rheb, an upstream activator of mTORC1 (Patel and Faesen, 2024). This not only shuts down energy-consuming anabolism but also clears the path for autophagy initiation.

Critically, AMPK and mTORC1 signaling converges on the ULK1 complex, a key node enabling precise control of its activity. AMPK can directly phosphorylate and activate ULK1, whereas mTORC1 exerts inhibitory phosphorylation. Thus, autophagy initiation is not merely a consequence of “mTORC1 turning off” but is dynamically driven by the dual signals of AMPK activation coupled with mTORC1 inhibition. Furthermore, AMPK can reinforce autophagic induction through mTOR-independent pathways, such as by phosphorylating Beclin-1 and FoxO3 (Ge et al., 2022; Al-Bari and Xu, 2020).

The activated variant of ULK1 can increase Beclin-1 activity, which is considered the mammalian counterpart of yeast Atg6 and is acknowledged as a pivotal marker in the early stages of autophagy. This interaction promotes the synthesis of phosphatidylinositol 3-phosphate (PI3P) through a complex that includes ATG14L, VPS15, and VPS34, thereby triggering the development of the autophagosome membrane. The VPS34 complex 1 is composed of VPS34, Beclin-1, ATG14L, and VPS15. ULK1 is essential for the phosphorylation of Beclin-1 phosphorylation, which subsequently leads to the activation of the VPS34 lipid kinase complex. Following its activation, VPS34 employs phosphatidylinositol (PI) as a substrate to generate PI3P (Vargas et al., 2023).

The ATG14L component facilitates the positioning of the VPS34 complex 1 to the preautophagosomal structure (PAS). Furthermore, PI3P serves to attract effector proteins, including the WD-repeat protein that interacts with PI3P (WIPI) and double FYVE domain-containing protein 1 (DFCP1). The VPS34 complex interacts with two ubiquitin-like protein-conjugating systems, specifically the ATG5-ATG12-ATG16L1 complex and ATG8/microtubule-associated protein 1 light chain 3 (LC3), via WIPI2. This interaction facilitates the extension of autophagosome membranes until they completely enclose the PAS, resulting in the formation of autophagosomes. The microtubule-associated LC3 protein is crucial in the process of autophagosome development (Glick et al., 2010).

After the cleavage of LC3 by the Atg4 enzyme, LC3I is generated. In the presence of the ubiquitinases Atg7 and Atg3, LC3I is converted into LC3II. LC3II interacts with phosphatidylethanolamine (PE) to create a complex that facilitates its action on the ATG12-ATG5-ATG16L1 assembly. This interaction plays a critical role in the elongation of the autophagosome membrane, ultimately leading to the formation of autophagosomes characterized by a double-layered membrane architecture. Beclin-1 and LC3II are upregulated upon enhanced autophagy and are markers reflecting levels of autophagy. The ratio of LC3II/I, called autophagic flux, is typically used in clinical practice to evaluate the intensity of autophagy in cells. After autophagosome maturation, the *in vitro* autophagic membrane of autophagy fuses with the lysosomal membrane through protein–protein interactions to form an autophagy-lysosome system. The protein LC3II interacts with the selective autophagy receptor, P62 (SQSTM1), facilitating the transport of autophagic materials into autolysosomes. Within these organelles, the contents are subsequently broken down by hydrolases into simpler compounds such as sugars, amino acids, and fatty acids, which can be recycled by the body’s cells. In instances where autophagy is inhibited, an increase in P62 protein levels is observed, and its expression is inversely related to LC3II/I and Beclin-1 (Islam et al., 2018).

Autophagy is orchestrated by highly integrated signaling networks that sense nutritional, energetic, and stress cues. Within the mTOR-centric network, the PI3K/Akt and MAPK pathways activate mTORC1 under nutrient-rich conditions by suppressing the TSC complex, subsequently phosphorylating ULK1 to block autophagy initiation; conversely, AMPK functions as an energy sensor that inhibits mTORC1 through phosphorylation of TSC2 and Raptor. Notably, AMPK can also directly phosphorylate ULK1 at Ser317 and Ser777, constituting an mTOR-independent activation mechanism.

At the parallel regulatory level, the Beclin-1-VPS34 complex is governed by competitive binding interactions with BCL-2 family proteins (inhibitory) and AMBRA1/UVRAG (activating), and is subject to direct phosphorylation by ULK1; meanwhile, p53 exhibits dual functionality, exerting pro-autophagic effects in the nucleus (transcriptional upregulation of DRAM1 and Sestrins) and anti-autophagic effects in the cytoplasm (sequestration of ATG7). Furthermore, stress-responsive pathways—including hypoxia (HIF-1α/BNIP3), endoplasmic reticulum stress (PERK/IRE1α), and calcium signaling (DAPK1)—mediate context-specific activation of autophagy through modulation of Beclin-1 activity or the ULK1 complex (Wen and Klionsky, 2020; Feng et al., 2018).

### 2.4 Autophagy and other types of cell death

Apoptosis is essential for the developmental trajectory of organisms and the preservation of environmental equilibrium in mature organisms. The elimination of superfluous cells during an organism’s developmental stages is a key process for ensuring normal morphogenesis and organ formation. In contrast, during adulthood, the clearance of autoreactive immune cells, cancerous cells, and damaged cells plays a vital role in maintaining internal homeostasis.

The regulation of cellular apoptosis is highly meticulous, with both excessive and insufficient cell death contributing to various human diseases (Liu et al., 2024). A pertinent illustration of this is the progression of neurodegenerative disorders, which frequently correlates with the premature demise of neurons that are typically expected to persist for an extended duration to uphold neurological functionality. Conversely, a hallmark characteristic of cancer is the body’s failure to adequately eliminate cells that harbor oncogenic mutations (Yuan and Ofengeim, 2024).

The Nomenclature Committee on Cell Death (NCCD) has established a set of guidelines to categorize cell death into various types. These classifications primarily include accidental cell death (ACD) and programmed cell death (PCD). The categorization is based on distinct morphological features, biochemical properties, and the biological implications associated with the process of cell death (Galluzzi et al., 2018). Among them, ACD is an unbiologically regulated death process that occurs when cells are subjected to accidental injury stimuli, such as necrosis (Peng et al., 2022a). In contrast, PCD is distinguished by the involvement of controlled signaling pathways that are crucial for the development of organisms and the regeneration of tissues. This includes various forms of cell death such as apoptosis, autophagy, pyroptosis, necroptosis, and ferroptosis (Zhu et al., 2024; Li et al., 2024).

The fundamental function of autophagy is to maintain cellular energy and metabolic equilibrium, in addition to providing a protective role. However, it is important to note that under specific circumstances, either the disruption or the overactivation of autophagic processes may result in apoptosis (Liu S. et al., 2023; Lin et al., 2024). Two main ways of cell death associated with autophagy have been identified.

The initial category is autophagy-dependent cell death (ADCD), which is characterized by a significant reliance on autophagic elements and is distinct from other programmed cell death modalities. A cellular process must satisfy several key criteria to be classified as ADCD. First, autophagic flux must be increased at the time of cell death. Second, the cell death process should be reversible when autophagy is inhibited genetically or pharmacologically. Third, at least two distinct autophagy-related molecules must be involved, and their combined activity—rather than individual action—should be necessary to execute cell death. Finally, the death process must occur independently of other known cell death mechanisms (Doki et al., 2022). The second mechanism is known as autophagy-mediated cell death (AMCD). In this process, autophagic molecules may engage in direct interactions with cell death-related molecules, potentially inducing apoptosis, necrosis, or ferroptosis through the dynamic functions of autophagy (Kang et al., 2022; Chen et al., 2024). The two types of cell death linked to autophagy are not completely autonomous and can occur simultaneously. Additionally, under certain conditions, the regulatory mechanisms driving the cell death process may shift between these two forms.

In recent years, autophagy-related cell death has been intensively reported, including ADCD, such as endoplasmic reticulum autophagy, mitophagy, and autophagic cell death, and AMCD such as apoptosis, necrosis, and ferroptosis, and these forms of cell death are achieved through direct interactions between autophagic molecules and cell death molecules or the dynamic regulation of autophagic function. Consequently, autophagy is essential for the regulation of the intracellular environment, and significantly influences cell survival and apoptosis (Zein et al., 2021).

## 3 Autophagy and eye disease

Ocular tissue is one of the tissues with the most active metabolism in humans (Yang Y. et al., 2024). The functions of the cornea, lens, trabecular meshwork, retina and optic nerve are highly dependent highly on cellular metabolic homeostasis and organelle integrity (Stepp and Menko, 2021). Autophagy plays a crucial role in preserving the physiological integrity of ocular tissues when confronted with external environmental factors, including ultraviolet radiation and infectious agents, as well as internal stresses such as oxidative injury and nutrient scarcity. This biological process facilitates the degradation of misfolded proteins within the corneal epithelial cells, eliminates mitochondria that have been compromised by photooxidative damage in retinal photoreceptor cells, and upholds the transparency of lens fibre cells (Klionsky et al., 2021). Nevertheless, dysregulated autophagy serves as a significant pathological mechanism in the progression of numerous ocular disorders. Consequently, this review intends to compile and organize numerous relevant studies concerning the modulation and therapeutic approaches related to autophagy in the progression of eye diseases. This synthesis is expected to provide fresh perspectives on the underlying mechanisms of ocular diseases and lay a solid theoretical groundwork for the development of therapeutic strategies aimed at targeting autophagy.

### 3.1 Age-related macular degeneration (AMD)

AMD is a progressive degenerative condition that impacts the macula of the retina, leading to irreversible central vision loss and the perception of distorted images, known as metamorphopsia, in those who are affected. This condition stands is a significant contributor to irreversible blindness among the elderly population worldwide (Kumar and Jurkunas, 2021). Although RPE dysfunction and choroidal neovascularization (CNV) are hallmark characteristics of AMD, the precise mechanisms underlying this form of macular degeneration remain elusive. In the later stages of the disease, AMD can be classified into two distinct types. Importantly, dry AMD accounts for approximately 80%–90% of all cases; in contrast, wet AMD is primarily accounts for the majority of vision loss (Hyttinen et al., 2021).

AMD is a complex condition influenced by multiple factors, incorporating significant genetic predispositions alongside various environmental risks. These environmental contributors encompass aging, tobacco use, atherosclerotic disease, obesity, hypertension, elevated cholesterol levels, and diets rich in fats (Kaarniranta et al., 2020). At present, the predominant therapeutic approaches for AMD predominantly target patients experiencing advanced stages of wet AMD. Nevertheless, there is a significant lack of irreversible treatment options that can effectively prevent the apoptosis and ensuing degeneration of RPE and photoreceptor cells (Nashine, 2021). The administration of intravitreal anti-vascular endothelial growth factor (anti-VEGF) injections has shown promise in suppressing CNV associated with wet AMD. However, this therapeutic approach is not without its challenges. Individuals diagnosed with neovascular AMD (nAMD) often necessitate these injections on a monthly or bimonthly basis indefinitely, presenting a significant burden for both patients and healthcare systems (Hollaus et al., 2022; Felszeghy et al., 2019).

**FIGURE 2:**
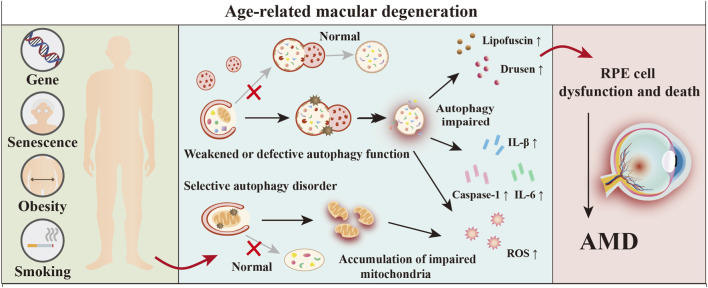
Core role of autophagy dysfunction in AMD pathogenesis. This schematic diagram summarizes the central role of dysfunctional autophagy in the pathogenesis of AMD. Genetic factors and environmental risk factors (such as aging, obesity, and smoking) collectively contribute to weakened autophagy and selective autophagy disorders in RPE cells. Impaired autophagy subsequently leads to the accumulation of intracellular waste, damaged mitochondria, exacerbated oxidative stress, and inflammasome activation. The accumulation of these cellular stress events ultimately triggers RPE cell dysfunction and death, thereby driving the onset and progression of AMD (Si et al., 2023).

AMD is predominantly characterized by degenerative changes in the retinal pigment epithelium, coupled with the buildup of lysosomal lipofuscin and the formation of extracellular drusen (Figure 2). The persistent oxidative stress, coupled with the aggregation of proteins and inflammatory responses, can result in the progression to geographic atrophy and/or the occurrence of choroidal neovascularization and subsequent fibrosis (Kaarniranta et al., 2023). RPE cells serves as a significant catalyst for the early onset of AMD, and autophagy is instrumental in the underlying mechanisms of this disease.

Autophagy plays a dual role by both removing intracellular metabolic waste and dysfunctional organelles, and influencing apoptosis pathways. A growing body of evidence indicates that the impairment of phagocytic activities and autophagic processes in RPE cells plays a significant role in the development of AMD. The evidence indicates that compromised autophagic activity is associated with the accumulation of lipofuscin granules and the presence of drusen in specimens obtained from *Nfe2l2/Nrf2* and *Ppargc1a/PGC-1α* knockout mice, as well as in RPE cells derived from human cadaveric cases of AMD (Kaarniranta et al., 2017). Orozco et al. employed an integrative analysis combining genomic and single-cell transcriptomic data to elucidate the genetic architecture of AMD. Their work not only corroborated 34 risk loci through genome-wide association studies but also pinpointed 15 putative causal genes including *TSPAN10 and TRPM1* several of which exhibit specific enrichment in the RPE, the primary site of AMD pathology (Orozco et al., 2020).

While RPE cells are crucial for clearing shed photoreceptor outer segments (POSs), photoreceptor cells themselves possess an active autophagic machinery essential for maintaining their health. These terminally differentiated neurons have high metabolic demands and are particularly vulnerable to proteostatic and oxidative stress. In AMD, autophagy dysfunction in within photoreceptors leads to the accumulation of damaged organelles and proteins, exacerbating cellular stress and contributing to their degeneration. This cell-autonomous defect in photoreceptor autophagy, coupled with the supporting RPE dysfunction, creates a vicious cycle that drives disease progression (Beatty et al., 2000).

RPE cells and photoreceptor cells display demonstrate considerable metabolic activity, which results in the production of significant amounts of reactive oxygen species (ROS) because of their high mitochondrial density (Song et al., 2017). Whereas autophagy regulates cellular antioxidant stress response via the *SQSTM1-KEAP1-NFE2L2/Nrf2* pathway. A decrease in autophagic function is associated with an increase in ROS levels, which subsequently triggers the activation of inflammasomes in RPE cells (Piippo et al., 2018). Autophagy significantly interacts with oxidative stress, while SQSTM1 plays a unique role in in connecting autophagic processes to the ubiquitin-proteasome system (UPS) (Demishtein et al., 2017).

The primary function of the antioxidant system relies on the efficacy of antioxidant enzymes and proteins that are integral to DNA repair processes. Additionally, autophagy interacts with antioxidant pathways via mechanisms related to the response to DNA damage (Czarny et al., 2015). Autophagy dysfunction within the RPE has been shown to intensify inflammatory reactions. In an *in vitro* setting, the levels of inflammasome-related caspase-1, as well as the cytokines IL-1β and IL-6, along with nitrite oxides and pro-angiogenic proteins, were markedly increased following the coculture of mouse RPE cells exhibiting abnormal autophagy with bone marrow-derived macrophages. These findings suggest that inflammasomes are activated following defective autophagy in the RPE (Liu et al., 2016). Autophagy has multiple regulatory function across various aspects, including maintaining the functional homeostasis of RPE, modulating cell death, counteracting oxidative stress, facilitating lysosomal activity, and influencing inflammatory responses. Specifically, it can increase cell survival while simultaneously triggering cell death, making it a critical element in the development of AMD.

In recent years, selective autophagy and secretory autophagy and their roles in AMD pathogenesis have received increasing attention. *BACE1* is also expressed in the retina, and its downregulation can lead to the upregulation of *PINK1* and *PRKN* expression in RPE cells under oxidative stress, indicating its potential impact on mitophagy (Francelin et al., 2021). In *Nfe2l2*/*Ppargc1a* double knockout mouse models, the expression of *PINK1* and *PRKN* was similarly found to be upregulated, accompanied by mitochondrial damage (Sridevi Gurubaran et al., 2020). The evidence suggests that there are interactions of autophagy,the endoplasmic reticulum, and mitochondria interact during the development of AMD in RPE cells. Nonetheless, impaired ER autophagy can result in the accumulation of improperly folded proteins and disrupt cellular functions, consequently worsening the pathology associated with AMD (Sun et al., 2021).

In AMD, secretory autophagy (SA), as an atypical secretory pathway, is involved in the physiological and pathological processes of cells. Secretory autophagy is involved in the inflammatory response, cellular senescence, and toxic protein accumulation processes in AMD by regulating the secretion of inflammatory factors such as IL1B, IL18, and IL33 (Cheng et al., 2021), toxic proteins such as Aβ (Liu et al., 2012), and ageing-related factors such as HMGB1 (Kim et al., 2021). The dysregulation of SA could potentially worsen the pathological progression of AMD, whereas targeting SA may represent a novel therapeutic approaches for managing this condition. In summarize, increased oxidative stress, protein misfolding, persistent inflammation, and choroidal neovascularization are implicated in the pathogenesis of AMD in certain individuals (Du and Palczewski, 2022).

MicroRNAs (miRNAs) also serve as potential therapeutic targets for modulating autophagy in patients with AMD. Studies indicate that multiple miRNAs are upregulated in individuals with AMD; however, conflicting results have been reported for certain miRNAs. For example, miR-34a expression is increased in the retinas of patients with AMD and in the plasma of wAMD patients with wAMD but is downregulated in the serum of patients with wAMD. Similarly, miR-29-3p expression is upregulated in the plasma of AMD patients but downregulated in choroidal/RPE tissues from wAMD patients. These discrepancies may arise from heterogeneity in sample sources, disease subtypes, detection methods, and other factors. Therefore, establishing standardized, tissue-specific miRNA expression profiles is crucial for elucidating the mechanisms of AMD and developing targeted therapeutic strategies (Hyttinen et al., 2021).

Sharma et al. generated clinical-grade iPSC-RPE patches free of oncogenic mutations from three AMD patients under GMP conditions; batches meeting the in-house thresholds (PEDF/VEGF ≥4, phagocytic index ≥75%, TER ≥250 Ω cm^2^, melanin OD480 ≥ 0.6) were loaded onto biodegradable scaffolds and transplanted into RCS rats and laser-injured pigs, yielding 68% a-wave recovery, 55% leakage reduction and 80% outer-nuclear-layer preservation without tumorigenicity—demonstrating that this quality-control-plus-delivery pipeline can provide a safe, effective and reproducible clinical template for autologous iPSC-RPE therapy of AMD (Sharma et al., 2019).

Under physiological conditions and in the early stages of AMD, autophagy protects retinal pigment epithelial cells by clearing waste, regulating antioxidant and anti-inflammatory responses, and cooperating with the ubiquitin-proteasome system. However, in advanced AMD, impaired autophagy leads to the accumulation of toxic substances, exacerbates oxidative stress and inflammation, and may shift toward pathological secretory autophagy and mitophagy. Therefore, autophagy in AMD is a dynamic system—its role, whether protective or pathogenic, depends on the integrity of autophagic flux, lysosomal function, and the state of the cellular microenvironment.

### 3.2 Diabetic retinopathy (DR)

DR is triggered by elevated blood glucose levels, which results in increased oxidative stress. This condition in turn initiates an adaptive inflammatory response within the microvascular structures and neuroretinal tissues (Saxena et al., 2016). Hyperglycaemia activates protein kinase C (PKC) (Idris et al., 2001), promotes the accumulation of advanced glycation end products (AGEs) (Stitt, 2010), activates the polyol pathway and the hexosamine pathway (Du et al., 2000), and leads to the overproduction of ROS, which in turn triggers oxidative stress and inflammation (Feng et al., 2007). This process, on the one hand, results in damage to the microvasculature, which is marked by the loss of pericytes, alterations in the blood-retinal barrier, and the obstruction of capillaries (Beltramo and Porta, 2013). On the other hand, retinal ischaemia leads to vascular VEGF overproduction through the activation of hypoxia-inducible factor-1 (HIF-1), which in turn triggers neurodegeneration, manifested as reactive gliosis, neuronal dysfunction, and neuronal apoptosis (Hammes, 2018).

**FIGURE 3:**
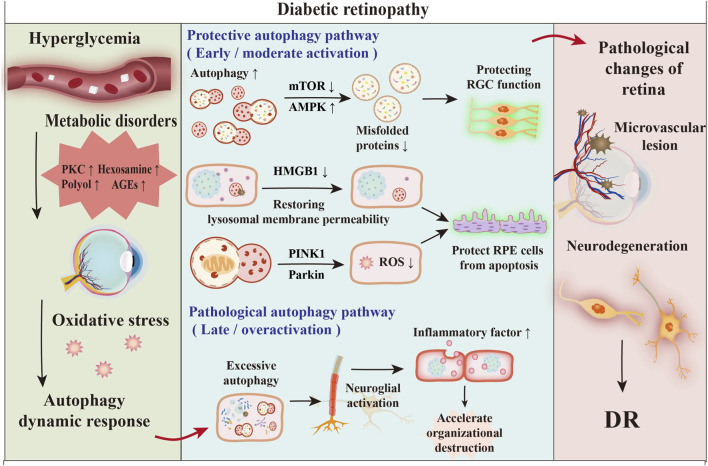
Autophagy plays a dual role in DR. Early hyperglycemia activates protective autophagy, clearing misfolded proteins via enhanced flux and mitophagy, thereby preserving retinal cell function. As DR progresses, excessive autophagy becomes pathological, promoting inflammation and tissue damage, ultimately driving microvascular lesions and neurodegeneration (Adornetto et al., 2021).

Microvascular injury and neurodegeneration work together to ultimately lead to the progression of diabetic retinopathy (Figure 3). Considering this evidence, the administration of intravitreal anti-VEGF agents is presently regarded as the benchmark for both early and advanced management of diabetic retinopathy. Other treatments include intravitreal steroids, laser photocoagulation, and vitreous surgery (Stitt et al., 2016). However, despite the clinical benefit shown by these treatments, there is no way to completely halt disease progression or reverse retinal damage. Indeed, these treatment modalities are typically employed during the advanced phases of diabetic retinopathy, characterized by an elevated risk of permanent and significant vision loss.

Recent studies suggest that autophagy is critically involved in the progression of diabetic retinopathy, demonstrating a robust association with the disease’s advancement as well as its possible reversal (Fu et al., 2016a). Autophagosomes present in cells play a vital role in the capture and breakdown of various biomolecules, including proteins, lipids, and entire organelles. Following this, they enable the transfer of these materials to lysosomes, where they are ultimately eliminated. This mechanism is essential for promoting the degradation of cellular components and aiding in the maintenance of metabolic processes. Furthermore, autophagy is integral not only to the quality control of cellular activities but also to the regulation of the degradation of cellular components in reaction to stress, thereby recycling essential nutrients necessary for sustaining cellular homeostasis (Yang et al., 2023).

In animal models, multiple research teams have confirmed the dynamic regulation of Atg in type 1 diabetes (T1D) and type 2 diabetes (T2D) (Piano et al., 2016). Notably, the protective role of autophagy in DR exhibits significant cell-type specificity. In retinal neurons such as ganglion cells, autophagy primarily exerts a clear neuroprotective effect by clearing toxic protein aggregates and damaged organelles, thereby countering high glucose-induced apoptosis (Solomon et al., 2017). In retinal vascular cells, however, its role is more complex: moderate autophagy contributes to the maintenance of vascular homeostasis, whereas excessive autophagy or the blockade of autophagic flux blockade under sustained stress may promote pericyte loss and compromise the blood-retinal barrier (Fu et al., 2016a). Thus, therapeutic strategies for DR that are designed to modulate autophagy may need to account for its differential effects on distinct retinal cell types.

Autophagy has been shown to have a protective function during the initial phases of DR. Mitophagy facilitates the elimination of oxidative damage-related byproducts, thereby mitigating oxidative stress (Santos et al., 2011). The downregulation of HMGB1 expression restores the integrity of lysosomal membranes, increases the efficacy of autophagic degradation, decreases the levels of VEGF and inflammatory mediators, and protects RPE cells from undergoing apoptosis in the early stages of DR (Feng et al., 2022). Delaying neurodegeneration protects RGC function by removing misfolded proteins.

Nonetheless, certain researchers have identified that the overactivation of autophagy serves as a significant pathological mechanism contributing to the progression of diabetic retinopathy. Silencing of the *HIST1H1C* gene, which is responsible for the production of histone H1.2, markedly suppressed both basal and glucose-stimulated autophagic activity. Additionally, this intervention led to a decrease in inflammatory responses and cytotoxic effects (Wang et al., 2017).

Conversely, the overexpression of *HIST1H1C* mediated by adeno-associated virus (AAV) promotes autophagy while simultaneously inducing glial activation and neuronal degeneration. Furthermore, recent research conducted by Madrakhimov et al. indicated that prolonged hyperglycaemia results in the inhibition of mTOR, subsequently leading to a disruption of autophagic processes. Notably, the administration of the mTOR activator MHY1485 effectively mitigated the impairment of neural cells in diabetic murine models through the inhibition of autophagy (Madrakhimov et al., 2021).

Moreover, certain researchers have observed the potential influence of a circadian rhythms on AGT protein concentrations. In contrast to previous studies, the retinas of mice with STZ-induced diabetes presented lower levels of Beclin-1, Atg7, p62, and LC3-II than those in the control group. Furthermore, the use of the heparanase inhibitor PG545 increased autophagy while concurrently decreasing the secretion of pro-inflammatory cytokines, thereby alleviating the symptoms associated with diabetic retinopathy (Qi et al., 2020). In a similar manner, the levels of Atg7, Atg9, LC3, and Beclin-1 were markedly decreased in the retinas of diabetic mice with streptozotocin (STZ)-induced diabetes, especially in the C57BL/6J mouse strain. This decrease was similarly observed in Bio-Breeding Zucker diabetic (BBZDR) rats, which are recognized for their spontaneous development of T2D, in comparison to their control counterparts (Mao et al., 2019).

These findings indicate that the molecular pathways governing basal autophagy could be modified in terms of both the strength and duration within the diabetic retina. DR has the potential to impair various retinal cellular components, including vascular pericytes, RPE, and ganglion cells. Consequently, numerous *in vitro* cellular studies have explored the mechanisms regulating autophagy across these distinct cell types in the context of diabetic retinopathy (Yang et al., 2023).

Kiamehr et al. undertook a research investigation aimed at exploring the impact of increased glucose concentrations, both with and without the presence of insulin, on cellular performance and the process of autophagy. This study employed a human induced pluripotent stem cell-derived retinal pigment epithelium (hiPSC-RPE) cell line sourced from individuals diagnosed with T2D alongside healthy control subjects. The findings demonstrated no significant difference in LC3-II expression between diabetic and healthy control cells, whereas a marked accumulation of p62 was detected in the diabetic cells. This increase in p62 expression may be linked to the activation of the antioxidant *NFE2L2-ARE* pathway, which is triggered by energy depletion in the cells affected by diabetes (Kiamehr et al., 2019). In the T2D mouse model, either hiPSC-CD34^+^ or hiPSC-ECFC alone improved retinal electrophysiological function, yet only hiPSC-CD34^+^ significantly restored retinal thickness. Their combined use produced a “non-additive” effect, pointing to a synergistic mechanism. RPPA profiling identified combination-specific alterations in PI3K-AKT-mTOR signaling, glycolysis, endothelial junction proteins, and m6A RNA methylation—nodes known to govern autophagy activity. Based on these data, we propose that co-delivery triggers network-level reprogramming of the “metabolism–epitranscriptome–autophagy” axis, culminating in synergistic repair of the diabetic retina (Calzi et al., 2025).

Autophagy occurs in retinal Müller cells. Studies have demonstrated exposure to high glucose concentrations leads to an increase in the initial phases of autophagy, as indicated by the elevated expression levels of Beclin-1 and LC3-II proteins. Conversely, the activation of autophagy by the mTOR inhibitor rapamycin increases Beclin-1 expression, mitigates the accumulation of p62 by facilitating the degradation of autophagic substrates, and protects cells from apoptosis (Lopes et al., 2016). Increased Atg5, Beclin-1, and LC3-II protein levels have been documented in human Müller cell lines (MIO-M1) that have been spontaneously immortalized and exposed to *in vitro* treatment with modified highly oxidized low-density lipoproteins (HOG-LDL). Furthermore, the use of 3-methyladenine (3-MA) to inhibit autophagy, coupled with the downregulation of Atg5 and Beclin-1, exert a partial protective effect on the apoptosis of Müller cells. These results indicate that autophagy plays a crucial role in the apoptotic process triggered by HOG-LDL (Fu et al., 2016b). Similarly, it has been found that autophagy also occurs in pericytes and plays a dual role: low levels of HOG-LDL promote pericyte survival and high levels lead to cell death (Proikas-Cezanne et al., 2015).

In addition, oxidative and glycated modified plasma lipoprotein extravasation is also an important driver of diabetic retinopathy, and its toxic effects on RPE cells may be associated with autophagy. These findings suggest a metabolic environment dependent effect on the regulation of autophagy in the retina (Mohammad and Kowluru, 2022). Mitophagy is an important type of selective autophagy in diabetic retinopathy, that is characterized by time and concentration-dependent biphasic regulation. Early compensatory activation delays pathological progression, whereas late dysfunction accelerates retinal damage. The PINK1/Parkin pathway is a core target for the regulation of mitochondrial mass, and its activity is significantly affected by ROS levels and sugar concentration gradients (Zhou et al., 2019).

Treatment with fenofibrate, a peroxisome PPARα agonist, prevented ER stress and induced autophagy and protected RPE cells in high glucose (25 mM, 18 days) and low oxygen (1% oxygen, 6 h or 24 h) environments, both of which are important components of the diabetic environment (Lazzara et al., 2020). Cai and colleagues demonstrated that male rats fed a diet high in fats and sugars showed an increase in LC3-II expression following an STZ injection. In this experimental context, the administration of glucagon-like peptide-1 (GLP-1) alleviated oxidative stress and inhibited the increase in LC3-II expression (Cai et al., 2017).

Autophagy plays a dual role in DR. In the early stages, it exerts protective effects by clearing oxidative damage, mitigating mitochondrial dysfunction, suppressing inflammation, and preserving the retinal pigment epithelium and ganglion cells. Under sustained hyperglycaemic stress, however, autophagy shifts towards a pathogenic mechanism, promoting inflammation, apoptosis, and vascular abnormalities. Selective autophagy, including mitophagy, displays biphasic regulation—compensatory regulation early on and dysfunctional regulation later—and is influenced by hyperglycaemia, oxidized lipoproteins, the cell type, signalling pathways, signaling pathways, and potential circadian rhythms. Thus, autophagy in DR functions as a context-dependent, dynamic regulatory system, supporting the rationale for stage-specific therapeutic targeting.

### 3.3 Glaucoma

**FIGURE 4:**
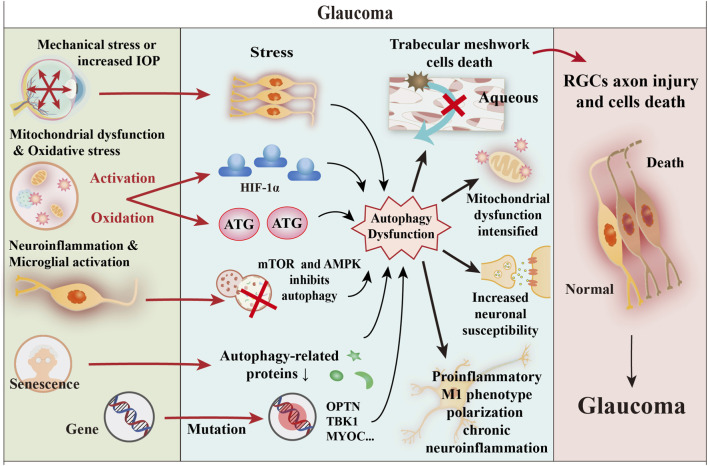
Core role of autophagy dysfunction in glaucoma pathogenesis. This figure illustrates the series of core mechanisms, initiated by mechanical stress or elevated IOP, that ultimately lead to RGC injury and death. The process begins with mitochondrial dysfunction and oxidative stress, which subsequently trigger neuroinflammation and microglial activation (Tribble et al., 2023). Concurrently, cellular autophagy is suppressed due to HIF-1α dysregulation, abnormal expression of autophagy-related genes, and imbalance in the mTOR/AMPK signaling pathway. These factors collectively contribute to the death of trabecular meshwork cells and, through a positive feedback loop, further exacerbate mitochondrial dysfunction. This results in chronic neuroinflammation and increased neuronal susceptibility, ultimately leading to the irreversible outcome of RGC axonal injury and cell death (FAN et al., 2022).

Glaucoma represents a prevalent ocular disorder that leads to blindness, marked by the gradual degeneration of RGCs and their associated axonal structures. This condition is additionally defined by the gradual decline in the integrity of the visual field, accompanied by the onset of optic nerve atrophy (Fan et al., 2022). Glaucoma is classified as a neurodegenerative disorder that is influenced by a variety of factors (Figure 4). The mechanisms driving glaucomatous neurodegeneration are not attributable to a singular factor; rather, they stem from the complex interplay and causal relationships among various elements. These include age-related increases in HIOP, oxidative stress, deficits in neurotrophic factors, mitochondrial dysfunction, autoimmune conditions, as well as genetic mutations (Stamer and Acott, 2012). In recent years, autophagy has attracted considerable attention as an essential process for maintaining neuronal equilibrium. Disruptions in autophagic activity have been associated with various neurodegenerative diseases (Tribble et al., 2023). Autophagy is essential in governing the onset and advancement of glaucoma, positioning it as a promising novel target for therapeutic interventions.

Increased intraocular pressure arises from TM/SC dysfunction, whereas autophagy serves as a critical adaptive mechanism for TM cells to cope with mechanical stress induced by this dysfunction (Shim et al., 2020). Under standard physiological conditions, autophagy induced by mechanical stretching is essential for the removal of impaired cellular components and significantly improves the recycling processes of proteins and organelles. This process contributes to the augmented stiffness of both the cytoskeleton and the cortical regions of trabecular meshwork cells, thereby supporting cellular homeostasis and preventing apoptotic cell death (Liton, 2016; Porter et al., 2014). Conversely, when autophagy is improperly regulated, it may trigger cellular death and exacerbate the advancement of various diseases, especially when mechanical stresses exceed normal physiological limits or arise in pathological contexts. Importantly, excessive activation of autophagy has been suggested as a possible cellular mechanism that plays a role in the optic nerve degeneration seen in DBA/2J mice (Hirt et al., 2018).

An additional significant source of ROS is the production facilitated by the Fenton reaction, which is catalysed by iron (Yauger et al., 2019). The increase in lipid peroxidation may be due to insufficient function of the antioxidant system (Fernández-Durango et al., 2008). ROS play a dual role in the regulation of autophagy. Autophagy is initiated by the activation of several transcription factors, including hypoxia-inducible HIF-1α, NRF2, p53, and FOXO3. The expression of genes essential for autophagy, such as *BECN1*, *LC3*, and *SQSTM1*, is subsequently modulated by posttranslational modifications. Conversely, the autophagic process can be suppressed by the oxidation of autophagy-related proteins, including ATG7 and ATG10, or through the inactivation of critical regulatory proteins associated with autophagy, such as transcription factor EB (TFEB) and phosphatase and tensin homologue (PTEN) (Chang et al., 2022).

On the other hand, autophagy serves to reduce ROS levels by removing dysfunctional molecules, including protein aggregates, as well as compromised organelles, such as mitochondria. This mechanism plays a crucial role in reducing oxidative damage, thereby maintaining the stability of redox signalling pathways and facilitating the repair processes of DNA damage (Li et al., 2015). These results indicate that the stimulation of mitophagy could enhance the pathogenesis of glaucoma. Consequently, mitophagy is significantly associated with oxidative stress and serves as a crucial selective form of autophagy involved in the progression of glaucoma.

In the pathological process of glaucoma, retinal injury and the establishment of a chronic inflammatory microenvironment—triggered by factors such as elevated intraocular pressure and oxidative stress—activate resident innate immune cells known as microglia. Activated microglia can polarize into either a proinflammatory phenotype (M1) or an anti-inflammatory phenotype (M2), a shift governed by changes in the microenvironment and referred to as classical activation and alternative activation, respectively (Lan et al., 2017). Chronic inflammation associated with glaucoma serves as a critical driver of RGC degeneration. Therefore, guiding microglial activation towards the neuroprotective M2 phenotype is of paramount importance (Quaranta et al., 2021). Autophagy acts as a molecular switch in this type of polarization: moderate autophagic flux promotes M2 conversion and releases anti-inflammatory mediators, whereas impaired or excessive shifts the balance towards M1, accelerating RGC apoptosis.

Autophagy plays a central regulatory role in this process. The proinflammatory cytokine TNF-α, which is released by M1 microglia, impedes autophagic flux in both neurons and microglia via the activation of the Akt/mTOR signalling cascade. This process, in turn, promotes the polarization of microglia towards the M1 phenotype (Jin et al., 2018). On the other hand, interleukin-4 (IL-4) promotes the differentiation of microglia into the M2 phenotype, increases the rate of autophagic flux, and has neuroprotective effects (Tang et al., 2019). M2 microglia are sustained through elevated levels of autophagic activity, whereas interference with this autophagic process leads to a transition towards the M1 phenotype. In conclusion, autophagy is essential in regulating inflammatory responses and offers protective advantages by facilitating the degradation of proinflammatory proteins (Zubova et al., 2022).

Autophagy is essential for the removal of superfluous proteins and cytoplasmic components. Nonetheless, the efficiency of this critical process often declines with ageing and the progression of neurodegenerative disorders (Mizushima et al., 2010). Studies have indicated that glaucoma, which is classified as a neurodegenerative disorder, is characterized by a decrease in the mRNA levels of autophagy regulatory proteins, including the ATG and Beclin proteins (Rodríguez-Muela et al., 2013). Impaired autophagy increases the susceptibility of aged mice (Ambra1 +/−) to axonal injury in optic nerve crush models (Bell et al., 2020). Additionally, the dysregulation of autophagy in trabecular meshwork (TM) cells, which is linked to an elevated intraocular pressure, also becomes more prevalent with age (Hirt et al., 2018). An increasing amount of research highlights the positive effect of autophagy on addressing the challenges associated with ageing and age-related disorders.

RGCs are key targets in glaucoma, and autophagy plays a pivotal role in their pathophysiology (Asrani et al., 2024). Elevated intraocular pressure disrupts axonal transport at the optic nerve head, triggering stress responses in RGCs and surrounding glial cells. In this context, autophagy is activated as a critical stress response mechanism (Tribble et al., 2023). However, its role in glaucomatous RGCs remains controversial and appears to be dual or even paradoxical. Both autophagy inducers (rapamycin) and autophagy inhibitors (3-methyladenine) have been reported to protect RGCs in different studies (Russo et al., 2018). Similarly, Atg4B deletion exacerbates RGC death in optic nerve injury models, but delays neurodegeneration in chronic glaucoma models such as DBA/2J mice (Dixon et al., 2023). Therefore, future therapeutic strategies should focus on fine-tuning the autophagic autophagic balance in RGCs, rather than simply promoting or suppressing autophagy overall. This approach offers a promising direction for neuroprotection aimed directly at preserving neuronal survival.

Glaucoma-associated gene mutations participate in distinct pathological stages of the disease by influencing autophagy pathways in specific cell types, demonstrating clear cell type specificity (Sears et al., 2019). The *MYOC* gene is predominantly expressed in trabecular meshwork cells. Its mutation can induce protein misfolding and endoplasmic reticulum stress, thereby impairing autophagic function and compromising aqueous humour outflow efficiency, thereby promoting the development and progression of elevated intraocular pressure (Kasetti et al., 2021; Yan et al., 2022). In contrast, the *OPTN* and *TBK1* genes are highly expressed in RGCs. The proteins they encode are key molecules for initiating selective autophagy, such as mitophagy. Loss-of-function mutations in these genes directly weaken the ability of RGCs to clear damaged organelles, increasing their susceptibility to pathological stress and thus playing a central role in the pathogenesis of normal-tension glaucoma and glaucoma-related neurodegeneration (Medchalmi et al., 2021; Tucker et al., 2014). These cell-type-specific pathological mechanisms provide an important theoretical basis for developing precise treatments targeting different subtypes of glaucoma.

*OPTN* is an autophagy receptor involved in selective autophagy and inflammatory regulation, and its mutations include *E50K* and *M98K*. The *E50K* mutation triggers retinal cell death by inhibiting autophagy, whereas the *M98K* mutation leads to abnormal transferrin receptor degradation and cell death by enhancing autophagy signalling (Zhang et al., 2021; Sirohi et al., 2013).On the one hand, *OPTN* mutation can cause block autophagic flux, and the accumulation of LC3-positive aggregates, and can trigger oxidative stress and mitochondrial dysfunction (Medchalmi et al., 2021).

Using a transgenic mouse model carrying the human *TBK1* gene (Tg-TBK1), Vessey et al. showed that the specific overexpression and aggregation of the TBK1 protein in RGCs leads to dose-dependent and progressive RGC loss, independent of elevated intraocular pressure. Based on the established roles of TBK1 in regulating autophagy and inflammation, its abnormal overexpression may ultimately mediate neurotoxicity through downstream mechanisms, including a disruption of the autophagy lysosomal pathway and aberrant activation of neuroinflammatory signalling (Fingert et al., 2017).

Changes in the expression of the *MYOC* gene result in disrupted autophagy mechanisms, which consequently trigger apoptosis in TM cells and contribute to elevated intraocular pressure. This phenomenon has been linked to the activation of a transcription factor known as CCAAT/enhancer binding protein homologous protein (Kasetti et al., 2021; Yan et al., 2022). Mutations in these genes interfere with autophagy through different mechanisms, which in turn promote the development of glaucoma.

Multimodal RNA-seq revealed that iPSC-derived ITGA6^+^ trabecular meshwork-like cells (iPSC-ITGA6^+^) possess a unique transcriptome and potently stimulate primary TM (pTM) cell proliferation while re-populating the TM and Schlemm’s canal in glaucoma models. Notably, the regenerative capacity of these cells is closely associated with the modulation of autophagy—a key cellular homeostasis process implicated in TM cell survival and function. Mechanistically, iPSC-ITGA6^+^ cells are enriched for the nuclear-paraspeckle-assembling lncRNA NEAT1.

Emerging evidence suggests that paraspeckles, through sequestering autophagy-related mRNAs or regulatory proteins, can finely tune autophagic activity. In this context, paraspeckle abundance directly correlates with the “rejuvenation” extent of resident pTM cells, potentially by enhancing autophagic flux and promoting the clearance of damaged organelles and proteins, thus improving cellular fitness. Boosting MENβ-associated-RNA-driven paraspeckle assembly amplifies this regenerative effect, likely through further coordinated activation of autophagy-related pathways, offering a cell-replacement-free, endogenous strategy for primary open-angle glaucoma (Feng et al., 2025).

Ageing is an important background driver of in the multifactorial aetiology of glaucoma. With advancing age, cellular autophagic activity generally declines, leading to the impaired clearance of toxic proteins and damaged organelles and a gradual disruption of intracellular homeostasis, which constitutes a key mechanism underlying neurodegeneration (Rodríguez-Muela et al., 2013; Hansen et al., 2018). Moreover, the age-related loss of trabecular meshwork endothelial cells and extracellular matrix remodeling contribute to trabecular meshwork sclerosis and increased resistance to aqueous humour outflow, results in elevated intraocular pressure. Persistently elevated intraocular pressure exerts mechanical stress on the lamina cribrosa and optic nerve head, directly damaging the axons of the optic nerve (Amadio et al., 2018).

Furthermore, ageing triggers a “bioenergetic crisis” in the optic nerve. On the one hand, cellular senescence and oxidative stress lead to mitochondrial dysfunction in RGCs, impairing energy metabolism. On the other hand, chronic neuroinflammation mediated by the senescence-associated secretory phenotype (SASP) not only accelerates extracellular matrix remodeling but also promotes synaptic degeneration and loss in RGCs (Ju et al., 2023; Zhang Y. et al., 2024). These mechanisms collectively act through both intraocular pressure-dependent and intraocular pressure-independent pathways, significantly increasing the vulnerability of the optic nerve and driving the progression of glaucoma.

### 3.4 Cataract

The lens is a unique capsular tissue with a structure consisting of an elongated clear fibrocyte core covered by cuboidal epithelial cells in the anterior layer. Lens transparency depends on the precise regulation of epithelial cell differentiation into organelle-free fibre cells. This differentiation process requires the concerted action of multiple signalling pathways, and transcriptional and translational mechanisms, and runs through the entire life cycle. If this differentiation process is disturbed (both during development and adulthood), it may lead to a misaggregation of lens proteins, which in turn triggers cataract formation (Bassnett, 2009; Wride, 2011). Cataracts continue to be the primary cause of visual impairment globally, although phacoemulsification along with intraocular lens implantation have emerged as a recognized and commonly employed therapeutic approach (Khairallah et al., 2015).

**FIGURE 5:**
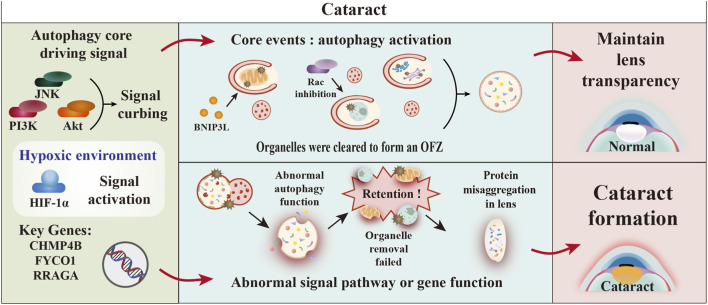
Core role of autophagy dysfunction in cataract pathogenesis. This diagram systematically illustrates the crucial role of the autophagy pathway in regulating lens transparency and the molecular mechanisms underlying cataract formation due to its dysregulation. Autophagy maintains lens transparency through coordinated organelle clearance mediated by signals including JNK, PI3K/Akt, and key genes (CHMP4B/FYCO1/RRAGA). This process creates the organelle-free zone via removal of mitochondria, ER/Golgi, and nuclei. Impaired autophagy due to signaling defects, gene mutations, or hypoxia causes organelle retention and protein aggregation, leading to cataract formation (Brennan et al., 2023).

Recent research indicates that autophagy plays a crucial role in the maturation of lens structures as well as in preserving homeostasis and transparency within the lens. A critical phase in this process is the transformation of lens fiber cells into organelle-free clear zones (OFZ) (Brennan et al., 2021). Research indicates that the removal of non-nuclear organelles, including mitochondria, the endoplasmic reticulum, and the Golgi apparatus within lens fiber cells, is facilitated by the signaling pathways MAPK, JNK, and PI3K, along with their respective downstream effector proteins such as Jun, Akt, mTOR, raptor, and p70S6K (Gheyas et al., 2022) (Figure 5). Inhibition of these signaling pathways is able to induce the expression of the autophagic essential molecule groups LC3-II and Beclin1, which initiate autophagosome-directed degradation of organelles and promote the formation of OFZ (Sue, 2022).

Utilizing *ex vivo* lens organ culture experiments, it was demonstrated that the suppression of JNK signaling resulted in the inhibition of mTOR and raptor phosphorylation within the mTORC1 complex. This disruption subsequently inhibited the activation of downstream p70S6K, which ultimately triggered the onset of autophagy. This mechanism promoted the breakdown of mitochondria, the endoplasmic reticulum, and the Golgi apparatus, while also aiding in the compaction and elimination of nuclear components. Inhibition of PI3K activity blocks activation of Akt and p70S6K, similarly leading to transformation of LC3B, thereby inducing the canonical autophagic pathway (Basu et al., 2014).

However, the inhibition of Akt alone, although capable of removing mitochondria and the endoplasmic reticulum, was not sufficient to eliminate nuclei. The elimination of the nucleus also requires the inhibition of Rac, which may involve the regulation of cytoskeletal or membrane dynamics and cooperate with autophagy to complete nuclear degradation (Gheyas et al., 2022). These results illustrate the existence of specific regulatory mechanisms for the clearance of different organelles.

The fusion machinery plays a central role in nuclear elimination in lens fibres. Lysosomal acid hydrolases, such as proteases, nucleases, and lipases, are released into the autophagic body cavity. These enzymes degrade the encapsulated nuclear material, including DNA and histone complexes, ultimately leading to nuclear clearance (Yang and Wang, 2021). This process reflects the critical function of the lysosomal enzyme system at the terminal stage of autophagy.

PLAATs (phospholipase A/acyltransferases) mark damaged mitochondria to initiate autophagic clearance by inducing the loss of the mitochondrial membrane potential (Morishita et al., 2021). Nevertheless, the formation of LAMP+ lysosomes, which is influenced by the differentiation status and modulated by the PI3K/mTORC1 signalling pathway, provides additional support for the accurate spatiotemporal regulation of nuclear and organelle degradation. This process provides a molecular basis for maintaining lens transparency. The nuclear excisome identified in primate lenses is a novel structure enclosed by a four-layered membrane and is directly involved in nuclear envelope degradation, which significantly advances our understanding of autophagy-mediated nuclear clearance (Costello et al., 2020).

The lens is one of the few tissues that operates in an oxygen-free environment, depending on the angle of light incidence and the diffusion of oxygen from the aqueous humour for its oxygen supply. Research conducted on oxygen levels in the lenses of both humans and chickens has confirmed the presence of an oxygen gradient within the lens, and a nearly 20% decrease in the oxygen concentration has been observed from the outer surface to the innermost core (Lutty and Mcleod, 2018; Beebe et al., 2014). Numerous active mitochondria located in the anterior epithelial layer mediate the continuous consumption of oxygen from the surrounding milieu, thereby creating a distinct metabolic gradient. When chicken embryonic lenses were cultured under either hypoxic or anoxic conditions (1% O_2_), the degradation of nonnuclear organelles clearly increased compared with that in lenses maintained under normoxic conditions (21% O_2_) (Bassnett and Mcnulty, 2003).

The hypoxic microenvironment promotes the upregulation of BNIP3L, a critical protein associated with mitophagy, through the activation of the HIF1α signaling pathway. This mechanism aids in the selective degradation of mitochondria, the endoplasmic reticulum, and the Golgi apparatus (Brennan et al., 2020). As a result, the process of autophagy induced by hypoxic conditions is crucial for the elimination of organelles that facilitate the development of the OFZ in the lens, in addition to its continuous role in the degradation of organelles during lens differentiation. If defects in BNIP3L or HIF1α function leads to organelle retention, lens opacification may be triggered, suggesting BNIP3L and HIF1α as potential targets for cataract therapy (Li et al., 2023).

Genetic research has revealed a significant correlation between mutations in autophagy-related genes and the occurrence of cataracts. Specifically, the gradual onset of posterior polar or subcapsular cataracts is associated with alterations in the *CHMP4B* gene (Shiels et al., 2007). *CHMP4B* functions as a fundamental component of the ESCRT-III complex, which plays a crucial role in the remodeling of cell membranes and is integral to various cellular division processes, including autophagy (Sagona et al., 2014). Mutations in the *CHMP4B* gene are associated with congenital and age-related cataracts, and their deletion leads to autophagosome accumulation, whereas the mutant affects the endosomal pathway and chromatin binding, thereby affecting degradation (Yonova-Doing et al., 2020; Radulovic et al., 2018).

Alterations in the *FYCO1* gene are linked to autosomal recessive congenital cataracts. The absence of FYCO1 results in a reduction in autophagosome formation, impaired autophagic activity, and the accumulation of organelles in differentiated fibrocytes. Changes in the *RRAGA* gene are associated with autosomal dominant congenital nuclear cataracts. Additionally, these mutations result in increased mTORC1 activity, which subsequently reduces autophagy levels (Xiao et al., 2021; Hong et al., 2017).

*TDRD7* functions as an essential component of RNA granules, significantly influencing RNA metabolism and regulation. Mutations occurring within *TDRD7* may interfere with the fusion process between autophagosomes and lysosomes, leading to an accumulation of autophagosomes and a disruption in the autophagic process. This sequence of events ultimately plays a role in the onset of cataracts (Zheng et al., 2014; Tu et al., 2021).

TBC1D20 regulates autophagosome maturation, whereas EPG5 is involved in autophagosome formation and clearance and participates in mutant genes important for cataract formation. These genes constitute a precise regulatory network that together maintain lens cell autophagy homeostasis, and their functional defects will become an important molecular basis for cataract development (Liegel et al., 2013; Sidjanin et al., 2016).

### 3.5 Keratoconus (KC)

**FIGURE 6:**
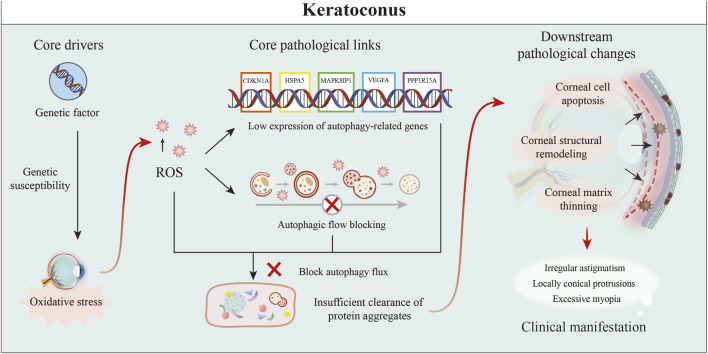
Core role of autophagy dysfunction in KC pathogenesis. Genetic predisposition and oxidative stress initiate the disease, driving ROS production. Core pathogenesis involves impaired autophagy from low expression of genes such as CDKN1A and HSPA5, which blocks autophagic flow and causes protein aggregation. This cascade triggers corneal cell apoptosis, stromal thinning, and structural remodeling, ultimately manifesting as the clinical hallmarks of conical protrusion, irregular astigmatism, and high myopia (Liu Y. et al., 2023).

KC is a corneal degenerative disease characterized by localized keratoconus-like projections, accompanied by thinning of the corneal stromal layer in the protrusion area, resulting in irregular astigmatism high myopia and in severe cases, blindness. The development of keratoconus is considered to be associated with genetic factors, familial predisposition, allergic conditions, and certain medical disorders. Nevertheless, its exact pathogenesis has not yet been fully elucidated (Naroo, 2008; Brookes et al., 2003). Autophagy, which is recognized as a crucial biological mechanism for sustaining cellular homeostasis and addressing stressors, has attracted increasing interest in recent research concerning KC (Figure 6).

Liu et al. performed an extensive bioinformatics investigation of autophagy-associated genes concerning keratoconus, employing data obtained from the GEO database. The study identified several critical autophagic genes, namely, *CDKN1A*, *HSPA5*, *MAPK8IP1*, *PPP1R15A*, and *VEGFA*, which exhibited markedly reduced expression levels in keratoconus tissues. Furthermore, these genes were found to be implicated in significant signaling pathways related to immune system disorders, circadian rhythm regulation, and metabolic processes. This study offers valuable insights into potential molecular targets and paves the way for novel research avenues aimed at the treatment of keratoconus (Yuan et al., 2024).

Kannan et al. identified 1,104 proteins by non-targeted proteomic analysis of tears from 40 healthy individuals and 69 KC patients (107 eyes, divided into Grades 1–4), 279 of which were quantifiable and 32 of which were significantly dysregulated in KC, and these differential proteins were enriched in pathways such as glycolysis, extracellular matrix (ECM) tissue, reactive oxygen species detoxification, and inflammatory regulation. The progression of KC has been identified as being linked to various biological processes, including neutrophil degranulation, autophagy, metabolic alterations, and protein phosphorylation. Consequently, the associated molecules and pathways have the potential to serve as promising therapeutic targets (Kannan et al., 2025).

Vottonen et al. identified 1,104 proteins by performing a nontargeted proteomic analysis of tears from 40 healthy individuals and 69 KC patients (107 eyes, divided into Grades 1–4), 279 of which were q quantified. They observed that the concentrations of 4-HNE, which serves as an indicator of lipid peroxidation, were approximately three times higher in the corneal basal epithelial cells of patients with KC than in those of healthy individuals. These observations indicate that oxidative stress may play a crucial role in the development of KC. Furthermore, the concentrations of the heat shock protein HSP70 and the protein aggregation indicator SQSTM1 were markedly increased, indicating a disruption of intracellular proteostasis, increased protein aggregation, and inadequate autophagic degradation. Activation of the autophagy signalling pathway is acknowledged as a vital mechanism for regulating immune responses. Furthermore, oxidative stress can affect the performance of immune cells by altering the expression of proteins associated with autophagy, indicating a potentially important pathological mechanism contributing to the development of KC (Shetty et al., 2017).

Shetty et al. identified 1,104 proteins by performing a nontargeted proteomic analysis of tears from 40 healthy individuals and 69 individuals with KC, and the results revealed a significant reduction in the levels of LC3-II and LAMP1 expression was observed in the pyramidal region of grade II and III keratoconus epithelium compared with that in the peripheral region. These observations suggest that impaired autophagy could be a pivotal factor in the development of keratoconus. Furthermore, an exploration of oxidative damage and molecular pathways associated with keratoconus revealed increased levels of the LC3-II and p62 proteins in HCECs subjected to hyperoxic conditions. Interestingly, the administration of chloroquine did not lead to a significant change in LC3-II levels, suggesting that oxidative stress might obstruct autophagic flux in HCECs (Shetty et al., 2017).

These results indicated that deficiencies in autophagy triggered by oxidative stress could play a role in the development of KC. Therefore, exploring individualized treatment strategies that combine multiomics data with autophagy indicators may lead to the selection of more effective treatment measures for future treatment options in the future.

### 3.6 Fuchs endothelial dystrophy (FECD)

FECD is a multifaceted hereditary condition that is associated with age-associated degeneration and primarily affects the corneal endothelium. It is defined as the presence of irregular localized thickening of Descemet’s membrane, bilateral asymmetric corneal oedema, a decreased density of corneal endothelial cells, and the accumulation of ECM (extracellular matrix) deposits. These pathological changes progressively culminate in a decrease in visual acuity (Kumar and Jurkunas, 2021). This condition represents the primary reason for corneal transplantation in Western nations, particularly among females. However, the implementation of this surgical intervention is constrained by a scarcity of donors and potential postoperative complications. Consequently, investigations of alternative therapeutic approaches are urgently needed (Zhang et al., 2023).

**FIGURE 7:**
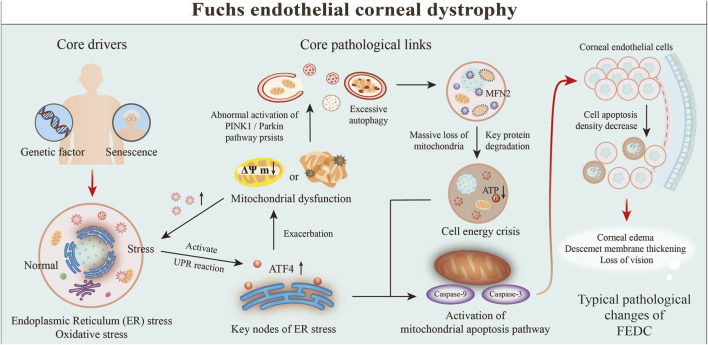
Core role of autophagy dysfunction in FECD pathogenesis. This illustration depicts the key pathogenic pathways in FECD. Genetic factors and cellular senescence trigger endoplasmic reticulum stress and oxidative stress, leading to mitochondrial dysfunction. This, in turn, induces excessive mitophagy and a cellular energy crisis, ultimately resulting in corneal endothelial cell death via the activation of apoptotic pathways. The cumulative effects of these processes manifest as the hallmark features of FECD: Descemet’s membrane thickening, corneal edema, and vision loss (Kumar and Jurkunas, 2021).

In the context of FECD, several elements lead to the aging of CEnCs (Figure 7). These factors encompass senescence triggered by stress, an imbalance between oxidants and antioxidants, damage and dysfunction of mitochondrial DNA, a prolonged unfolded protein response (UPR), and stress experienced within the ER. A transcriptomic meta-analysis has indicated that ERRα oxidative phosphorylation is a characteristic feature in the context of FECD. Notably, autophagy emerges as a crucial mechanism for maintaining the homeostasis of CEnCs and for their adaptive responses to cellular stressors. This autophagic pathway effectively facilitates the elimination of damaged cellular components, thereby enhancing cell survival in the face of oxidative stress and various other stress conditions. Given that mitochondria serve as the energy production centers within cells, any alterations in their bioenergetics and dynamics can have a profound impact on both the viability and functionality of CEnCs (Kumar and Jurkunas, 2021; Méthot et al., 2020). Numerous investigations have established that mitochondrial impairment and the process of mitophagy play crucial roles in the progression of FECD, particularly in relation to reduced viability of endothelial cells.

Using L450W and Q455K knock-in mouse models of *Col8a2* to investigate FECD, Meng et al. found that both models recapitulated the features of the disease, but the *Col8a2/Q450W/Q450W* mice presented less severe symptoms. This investigation highlighted the activation of the UPR in both models, as demonstrated by the enlargement of the rough endoplasmic reticulum and the increased expression of associated genes and proteins. Importantly, in CEnCs derived from mice aged 40 weeks, the expression of *Dram1* expression was increased by factors of 2.1 and 5.2, respectively. Similarly, a 10.4-fold increase in DRAM1 expression was observed in patients with FECD compared with control individuals. These findings suggest that the improper regulation of autophagy could play a significant role in the progression of FECD (Meng et al., 2013).

Benischke et al. identified the atypical activation of mitophagy in the corneal endothelial cells of individuals diagnosed with FECD. This particular anomaly was marked by a reduced mitochondrial membrane potential, a decrease in the mitochondrial mass, a partial breakdown of the electron transport chain complexes, and increased expression of autophagy indicators, including LC3 II and LAMP1. Additionally, electron microscopy revealed autophagic vacuoles that contained deteriorated mitochondria. Furthermore, *in vitro* experiments indicated that mitochondrial depolarization induced by carboxycyanide m-chlorophenylhydrazone (CCCP) resulted in significant decreases in both mitochondrial mass and the levels of Mfn2. Furthermore, the application of the mitophagy inhibitor bafilomycin restored the mitochondrial mass and MFN2 levels. These results indicate that the observed mitochondrial depolarization and subsequent dysfunction in FECD cells initiate mitophagy, leading to the degradation of MFN2 through the mitophagy pathway instead of through the traditional ubiquitin-proteasome system (Benischke et al., 2017).

Miyai et al. found significant accumulation of PINK1 and phosphorylated Parkin (Ser65) in corneal endothelial cells from FECD patients, accompanied by reduced Drp1, mitochondrial fragmentation, and autophagosome formation (Miyai et al., 2019). *In vitro* experiments showed that MN, an oxidative stress inducer, activated mitophagy and promoted PINK1/Drp1 degradation through Parkin, while CCCP activated Parkin without this effect, suggesting that oxidative stress overactivated abnormal mitophagy through the PINK1-Parkin axis. Liu et al. demonstrated that LYC significantly increased the expression of P62, thereby stimulating autophagy. This activation facilitated the degradation of Keap1 and promoted the nuclear translocation of Nrf2, ultimately leading to improved antioxidant capabilities and a decrease in apoptosis. The observed effects were facilitated by the regulation of the P62-autophagy-Keap1/Nrf2 signaling pathway (Liu C. et al., 2025).

Qureshi and colleagues observed a significant upregulation of the endoplasmic reticulum stress signalling pathway (PERK-eIF2α-ATF4-CHOP) and an increase in the expression of mitochondrial apoptotic indicators (cleaved PARP and caspase 9/3) in the Fuchs corneal endothelial cell line (F35T) when assessed under standard conditions. These changes were also associated with a reduction in mitochondrial membrane potential (MMP) as well as heightened fragmentation. Treatment with tunicamycin intensified these observed phenotypes. The siRNA-mediated silencing of *ATF4* using siRNA resulted in a reduction in ER stress and mitochondrial dysfunction, increased the levels of Mfn2/Tim23, and inhibited the excessive mitophagy mediated by Parkin, along with the activity of the Akt/mTOR pathways, which is related to autophagy. Additionally, compared with control mice, *Atf4* ± mice presented increased numbers of corneal endothelial cells, more regular cellular morphology, and decreased expression of CHOP following UVA irradiation. These results highlight the essential function of ATF4 as a key element in the relationship between the endoplasmic reticulum and mitochondria, thus providing a foundation for prospective targeted treatment approaches (Liu C. et al., 2025).

Corneal endothelial cells contain a large number of mitochondria is essential for maintaining corneal transparency. Although the above studies suggest that autophagy regulation is a potential therapeutic direction for FECD, the specific intervention strategies used to enhance or inhibit autophagy are still controversial, and further mechanistic exploration and clinical validation are urgently needed.

### 3.7 Herpes simplex keratitis (HSK)

**FIGURE 8:**
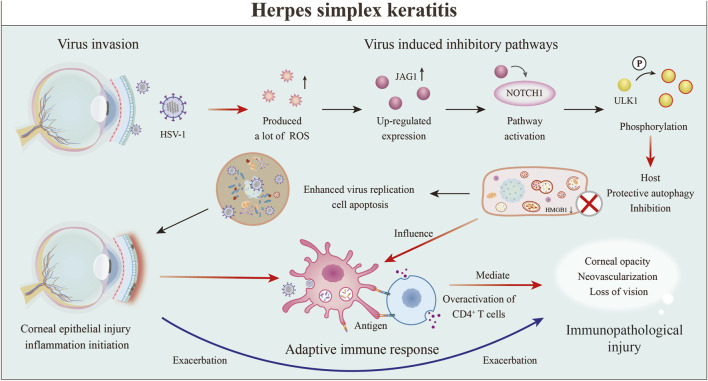
Core role of autophagy dysfunction in HSK pathogenesis. HSV-1 corneal infection inhibits host protective autophagy through the JAG1/NOTCH1/ULK1 pathway and induces ROS production, promoting viral replication and apoptosis. This initial damage triggers an adaptive immune response where dendritic cell-mediated CD4^+^ T cell overactivation drives immunopathology, including corneal opacity and neovascularization, ultimately leading to vision loss (Chang et al., 2024).

Herpes simplex keratitis (HSK) is caused by herpes simplex virus (HSV) infection and is classified into two types based on the viral subtype: HSV1 and HSV2, with HSV1 being the predominant pathogenic subtype. Studies have shown that during replication, HSV1 interferes with the host cell autophagy machinery, which normally plays a key role in clearing viruses and damaged cellular components. This interference allows the virus to evade intracellular clearance, thereby establishing persistent infection in corneal epithelial cells (Yakoub and Shukla, 2015) (Figure 8).

Yakoub et al. treated human HCECs with the proteasome inhibitor MG132 to investigate its regulatory role in autophagy. The results indicated a notable increase in the levels of LC3-II and significant decrease in SQSTM1 levels, thereby providing strong evidence for the successful initiation of the autophagic process. Moreover, upon subjecting both MG132-treated and untreated HCECs to infection with the HSV1-RFP virus, the flow cytometry analysis revealed a notable decrease in both the percentage of HSV1-RFP-positive cells and the mean fluorescence intensity in the MG132-treated cohort. These observations provide direct evidence that the stimulation of autophagy can substantially impede both the infection and replication of HSV1 in HCECs (Yakoub and Shukla, 2015).

Chang et al. found that HSV1 infection induced excessive ROS production in CECs, resulting in upregulation of *JAG1* expression and activation of the JAG1/NOTCH1 signaling pathway, and then NOTCH1 inhibited autophagy by promoting ULK1 phosphorylation (pULK1), as shown by reduced LC3A/B transformation, p62 accumulation and decreased autophagosome number. This reversal occurs when the JAG1/NOTCH1 signaling pathway is inhibited, for instance, with the use of DAPT, or when autophagy is activated, such as through rapamycin treatment. These therapeutic approaches result in a diminished accumulation of ROS and a reduction in apoptotic cell death. Concurrently, inflammation *in vivo* was observed to elevate the expression of *JAG1* in the corneal epithelium of HSK mice. Notably, either the suppression of *JAG1* or the promotion of autophagy has been shown to alleviate corneal damage, mitigate inflammatory cell infiltration, and reduce epithelial apoptosis. Therefore, targeting regulation of ROS/JAG1/NOTCH1/pULK1 pathway or activation of autophagy may become a new therapeutic strategy for HSK (Chang et al., 2024).

Jiang et al. determined that HSK is a condition characterized by ongoing inflammation mediated by CD4+ T lymphocytes. They also identified that the process of autophagy in dendritic cells plays a crucial role in the development of HSK. In experiments utilizing a mouse model that exhibited a deficiency in dendritic cell autophagy, it was observed that the activation of CD4+ T was diminished, which subsequently led to a marked reduction in both corneal opacity and neovascularization (Jiang et al., 2015).

Moreover, Grinage et al. reported that infection with HSV1 can induce diseases affecting both the ocular systems and nervous systems. They highlighted the importance of the host protein OPTN, which functions as a selective autophagy receptor, in providing crucial protective mechanisms that restrict HSV1 replication. This restriction not only inhibits the transmission of the virus to the central nervous system but also mitigates neuronal damage by facilitating the degradation of viral proteins, stimulating the production of type I interferons, and curtailing excessive inflammatory responses. These findings indicate that OPTN may serve as a promising target for therapeutic intervention (Grinage and Shukla, 2022).

In summary, the interplay between autophagy and viral infections represents a multifaceted phenomenon characterized by a dynamic equilibrium between the host and the virus. Subsequent investigations are crucial to comprehensively understand the specific mechanisms by which autophagy operates in different viral infections, including but not limited to HSV1. This exploration could reveal novel targets and approaches for the development of antiviral treatments.

### 3.8 Dry eye

**FIGURE 9:**
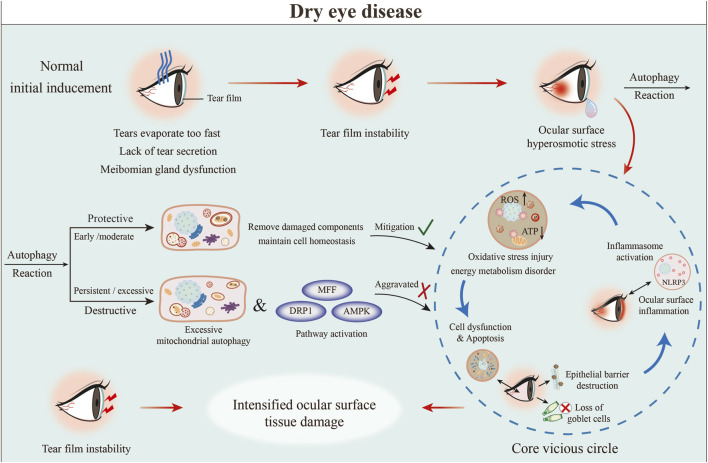
Core role of autophagy dysfunction in DED pathogenesis. Initial triggers like tear deficiency cause hyperosmotic stress, inducing autophagy. While moderate autophagy is protective, excessive AMPK/MFF-mediated mitophagy causes mitochondrial dysfunction, oxidative stress and energy deficit. This promotes cell apoptosis, ocular surface inflammation, goblet cell loss and epithelial barrier disruption -forming a key vicious cycle in dry eye progression (Ma et al., 2019; Peng et al., 2022b).

Dry eye disease is characterized by immune-mediated chronic inflammation, and inflammation is the core mechanism underlying its development. Inflammatory responses on the ocular surface constantly activate immune cells, especially T cells. These activated immune cells trigger excessive inflammation and oxidative stress, causing apoptosis in cells on the ocular surface cell apoptosis and glandular and corneal nerve damage, aggravating tear film instability and tear hyperosmolarity to result in a vicious cycle. While autophagy is an intracellular process that affects cell survival, its role in regulating the process of immune inflammation has been extensively studied, and it also plays an important role in the pathogenesis of dry eye (Figure 9).

At present, autophagy has a “bidirectional” nature in dry eyes. On the one hand, activating autophagy can help restore ocular surface metabolic homeostasis and reduce inflammation and oxidative stress damage; on the other hand, excessive autophagy can also mediate cell death and aggravate ocular surface cell damage, thereby promoting the progression of dry eyes. Liu et al. established a dry eye model by inducing primary HCECs in hypertonic medium and found that hypertonic stimulation induced inflammatory mediator expression and promoted upregulation of autophagy-related gene expression, and autophagy activation was later than inflammatory induction (Liu et al., 2020a).

Ma et al. found a mild increase in corneal autophagy levels in a dry eye C57BL/6 mouse model, and after intervention using autophagy activator (LYN-1604) and inhibitor (3-methyladenine), confirmed that activation of autophagy reduced dry eye parameters such as ocular surface inflammation, improved tear secretion and corneal staining, while inhibition of autophagy had the opposite effect (Ma et al., 2019). Wang and colleagues discovered that an increased level of DNA Damage-Inducible Transcript 4 (DDIT4) on the ocular surface initiated oxidative stress, subsequently resulting in compromised autophagy and reduced cell viability in corneal epithelial cells. Furthermore, the silencing of *DDIT4* expression reinstated typical autophagic activity and mitigated the associated damage (Wang et al., 2019).

Additionally, an investigation carried out by Stuard et al. revealed that corneal epithelial cells can secrete insulin-like growth factor binding protein 3 (IGFBP-3). This protein is essential for the regulation of metabolic homeostasis, helping to restore balance in response to hypertonic stress conditions. The protective effects observed in the corneal epithelium are intricately linked to the processes of mitophagy mediated by BNIP3L/NIX and MFN2. These findings were further corroborated in conjunctival epithelial cells (Stuard et al., 2022; Bogdan et al., 2021).

Peng et al. discovered that high osmolality (HOP) triggers oxidative mitochondrial damage and stimulates mitochondrial division and mitophagy through the AMPK/MFF pathway in HCECs. The knockdown of *AMPK* or *MFF* alone decreases HOP-induced mitochondrial division and autophagy, and also reverses mitochondrial fragmentation and mitophagy (Peng et al., 2022b). Using a mouse dry eye model, Xie et al. showed that chlorogenic acid (CGA) significantly improved ocular surface signs, reduced meibomian gland inflammation, and decreased oxidative stress markers by regulating the AMPK/ULK1 signalling pathway and inhibiting cellular autophagy and apoptosis (Chen et al., 2025). These findings indicate that in the context of dry eye disease, inhibiting autophagy may also restore cellular homeostasis and produce therapeutic effects. These findings suggest that future treatment strategies should focus on precisely regulating the “extent” and “timing” of autophagy, rather than simply activating or inhibiting it in a broad, nonspecific manner.

Studies on the use of autophagy modulators in the treatment of dry eye are emerging; for example, rapamycin is an autophagy activator that inhibits mTOR, and Cho et al. treated ageing mice with rapamycin eye drops, and the results showed that rapamycin improved symptoms such as ocular surface inflammation and goblet cell loss caused by ageing (Cho et al., 2019). The team further tried to use materials such as polyethylene glycol to encapsulate rapamycin to formulate eye drops for treatment and observed that treatment with rapamycin eye drops treatment could relieve lacrimal gland inflammation, increase tear secretion and improve the ocular surface status by upregulating ULK1 expression (Shah et al., 2017).

Trehalose, recognized as a non-reducing disaccharide, is noted for its potential role as an autophagy inducer and is employed as a bioprotective agent. Current studies demonstrate that eye drops fortified with trehalose can significantly maintain the viability of human corneal epithelial cells exposed to desiccation stress. Functioning as an autophagy enhancer, trehalose diminishes inflammatory responses by promoting autophagic flux through specific transcriptional pathways. Moreover, trehalose mitigates inflammation on the ocular surface through the activation of autophagy while concurrently inhibiting the MAPK signaling pathway. This action involves trehalose facilitating the nuclear translocation of TFEB, thereby augmenting autophagic flux and mitigating the inflammatory response associated with dry eye conditions (Panigrahi et al., 2019; Liu et al., 2020b).

Based on a systematic review of 10 randomized controlled trials (RCTs) published before 8 August 2023, trehalose artificial tears have demonstrated significant efficacy and safety in the treatment of DED. Compared with control groups, trehalose treatment showed superior improvement across all evaluated indicators, including significant reductions in the Ocular Surface Disease Index (OSDI) score, prolonged tear breakup time (TBUT), increased tear film thickness (TFT) and tear meniscus height (TMH), as well as improvements in Schirmer test results, corneal fluorescein staining (CFS), and visual acuity. No adverse events were reported during the treatment. The conclusion indicates that trehalose artificial tears are a safe and effective treatment for dry eye disease, and are particularly recommended for use in the preoperative period of cataract surgery (Ballesteros-Sánchez et al., 2023).

Calcitriol is the active metabolite of vitamin D3 and has anti-inflammatory and antiapoptotic effects. Lyu et al. showed that treatment with calcitriol can reduce pathological damage caused by apoptosis in dry eye disease by activating the vitamin D receptor (VDR) signalling pathway to increase autophagic flux (Lyu et al., 2020).

## 4 Therapeutic perspectives and challenges

Recently, the crucial role of autophagy in the progression of numerous eye diseases has been increasingly recognized, emphasizing its promise as a feasible target for therapeutic intervention. Nevertheless, this area of research is not without its challenges. As previously discussed, autophagy clearly presents unique therapeutic opportunities for different eye diseases. Prominent instances include the removal of impaired mitochondria, the disintegration of protein aggregates such as lipofuscin linked to AMD, and the alteration of inflammatory pathways, especially via the inhibition of the NLRP3 inflammasome in DR. As we increase our understanding of the underlying mechanisms of autophagy, the potential for creating targeted treatments to address these widespread ocular conditions continues to increase.

Autophagy is essential for maintaining the functional equilibrium of RPE cells, as it promotes the elimination of lipofuscin and damaged organelles. Furthermore, it bolsters cellular defence against oxidative stress through the SQSTM1-KEAP1-NFE2L2/NFE2L2 signalling cascade, modulates inflammatory responses by preventing inflammasome activation, and maintains the equilibrium of apoptosis. Impaired autophagy is a major factor contributing to the development of AMD (Hyttinen et al., 2019). In addition, defects in mitophagy, ER autophagy and abnormal secretory autophagy can also lead to important factors associated with retinal cell injury. Therefore, targeted repair of autophagy function in RPE cells to enhance antioxidant and anti-inflammatory effects and delay AMD progression is an important research direction. Drugs targeting autophagy-related pathways, such as *NFE2L2/Nrf2* and *PPARGC1α*, increase autophagic activity, thereby reducing oxidative stress and inflammation (Nita and Grzybowski, 2023). Furthermore, therapeutic genes are being delivered into RPE cells using adeno-associated virus (AAV) vectors (Cai et al., 2025). This approach aims to enable the continuous secretion of anti-VEGF antibodies or other therapeutic proteins, with the expectation of goal achieving a long-lasting therapeutic effect from a single injection (Osborne et al., 2018). This advancement holds promise for more effective and convenient treatment strategies in the management of eye diseases.

This study conducted a systematic review and quantitative meta-analysis to examine the association between oral metformin use and the risk ofAMD. Based on a comprehensive analysis of nine observational studies involving approximately 1.427 million diabetic patients, the results indicated that oral metformin use was associated with a lower odds ratio of AMD in patients with type 2 diabetes, reaching 0.63 (p = 0.004). However, some of the included studies presented conflicting conclusions regarding the relationship between total metformin exposure and AMD risk. These findings suggest that metformin holds significant potential in the treatment of AMD, though the association between metformin and AMD risk still requires further validation through prospective studies (Vessey et al., 2024).

Sudden gene therapy for DR is closely related to oxidative stress, inflammatory response and microvascular and neuroretinal injury caused by hyperglycemia. It is difficult to completely block the course of the disease or reverse the injury by current treatment. Autophagy presents a bidirectional effect dependent on stage, cell and stimulation intensity in DR. It plays a protective role by removing injurious substances in the early stage, and excessive activation in the late stage exacerbates the pathological process, which is closely related to a variety of retinal cell functions (Rosa et al., 2016). Recent research has revealed that targeting pivotal autophagy pathways, such as PINK1/Parkin-mediated mitophagy and mTOR-HMGB1-regulated lysosomal function, as well as employing combined metabolic interventions like PPARα agonists and GLP-1 analogues, demonstrates significant therapeutic potential (Zhang S. et al., 2024).

Anti-VEGF therapy holds significant importance for conditions characterized by neurodegeneration, particularly due to the gradual degeneration of retinal ganglion cells. The underlying mechanisms contributing to these diseases are complex and include a variety of factors, such as elevated intraocular pressure, oxidative damage, dysfunction in microvasculature, and genetic susceptibilities (Chang et al., 2022). In an RPE endothelial co-culture model, rapamycin achieved superior CNV suppression to anti-VEGF alone: it simultaneously reduced VEGF production by RPE cells and blunted endothelial responsiveness to VEGF, markedly decreasing sprouting in the “anti-VEGF non-responder” group without inducing apoptosis (Stahl et al., 2008). In a rat model of pathological retinal neovascularization, treatment with the mTOR inhibitors rapamycin and everolimus reduced both neovascular tuft area and downstream mTOR signaling; however, both agents also enlarged the avascular zone. With respect to the treatment ofnAMD, rapamycin has also been used in conjunction with anti-VEGF therapy, aflibercept (Yagasaki et al., 2014).

In a small clinical trial, intravitreal rapamycin combined with aflibercept, compared to aflibercept alone, administered every 2 months over 36 months did not yield significant improvements in central subfield thickness or best-corrected visual acuity. The potential anatomical benefits of this therapy require confirmation through larger cohort studies to establish their clinical significance (Rowe et al., 2023).

In the DR model, either hiPSC-CD34^+^ or hiPSC-ECFC alone improved retinal electrophysiological function, yet only hiPSC-CD34^+^ significantly restored retinal thickness. Their combined use produced a “non-additive” effect, pointing to a synergistic mechanism. RPPA profiling identified combination-specific alterations in PI3K-AKT-mTOR signaling, glycolysis, endothelial junction proteins, and m6A RNA methylation—nodes known to govern autophagy activity. Based on these data, we propose that co-delivery triggers network-level reprogramming of the “metabolism–epitranscriptome–autophagy” axis, culminating in synergistic repair of the diabetic retina.

Autophagy is essential in the pathological processes associated with glaucoma, as it aids in maintaining cellular equilibrium and safeguarding ganglion cells. However, it may also exacerbate injury when excessively activated (Kitaoka and Sase, 2023). Autophagy can stabilize intraocular pressure by regulating autophagy and primary cilia-autophagy pathway in trabecular meshwork cells, induce mitophagy to relieve oxidative stress and mitochondrial damage to protect retinal ganglion cells, regulate autophagic flux to balance microglial polarization to reduce neuroinflammation, and can also repair autophagic function against *OPTN*, *TBK1*, *MYOC* (Zhang et al., 2021) and other gene mutations.

CRISPR-Cas9 knockdown of mutant *MYOC* in human trabecular-meshwork cells and POAG mice relieves ER stress, normalizes autophagic flux, and lowers IOP, halting subsequent optic-nerve damage; feasibility in intact human eyes has been validated in an *ex vivo* organ-culture platform (Jain et al., 2017). TBK1 protein kinase, which mediates E50K-OPTN and M98K-OPTN induced cell death, is emerging as a potential drug target (Swarup and Sayyad, 2018). Using 661W photoreceptor-like cells stably expressing M98K-OPTN, study demonstrated that the mutant sensitizes retinal cells to ER stress and TNFα-induced apoptosis and that PERK/IRE1α upregulation is autophagy-dependent (Sayyad et al., 2023). These approaches provides a validated cell-based platform for dissecting how OPTN polymorphisms impair autophagy–ER stress crosstalk in glaucoma.

Cataracts continue to be the foremost cause of blindness globally, and their development is significantly associated with impaired autophagic processes in lens cells. The clarity of the lens depends on the precise elimination of organelles such as mitochondria, the endoplasmic reticulum, and the nucleus throughout the differentiation of fibre cells (Brennan et al., 2023). Autophagy induction facilitates the elimination of these organelles by modulating the MAPK/JNK/PI3K-mTORC1 signalling cascade, thereby ensuring that lens remains clear and functional (Gheyas et al., 2022). An oxygen gradient inside the lens, and the hypoxic microenvironment drives selective autophagy in organelles such as mitochondria through the HIF1α–BNIP3L pathway, which is an important for guaranteeing for OFZ formation; thus, targeting *HIF1α*-*BNIP3L* to increase hypoxia-induced autophagy activity can repair organelle clearance defects and provide a new strategy for preventing or reversing lens opacification (Li et al., 2023). Mutations in various autophagy-related genes such as *CHMP4B*, *FYCO1*, *RRAGA*, and *TDRD7*, disrupt their functions, disrupt their functions, resulting in impaired autophagic flow and the development of congenital or age-related cataracts (Radulovic et al., 2018).

Currently, the clinical management of cataracts primarily relies on surgical intervention. However, mechanism-based research is increasingly exploring aetiological therapies targeting autophagy. By correcting genetic defects or modulating key autophagic pathways such as HIF1α–BNIP3L, it is possible to restore autophagic homeostasis and organelle clearance function can be restored in the lens (Brennan et al., 2023). Therefore, correcting the function of these genetic mutations can improve autophagy homeostasis at the source and delay or treat cataracts.

KC is an eye disorder that results in visual dysfunction, marked by a decrease in the thickness of the corneal stroma and cone cells. The pathophysiology of this disease is intricately linked to autophagy dysfunction. Studies suggest that reduced expression of essential autophagy-related genes, such as *CDKN1A* and *HSPA5*, in conjunction with compromised autophagic flux contribute notably to the development of this condition (Yuan et al., 2024). Proteomic investigations have revealed that disease progression is correlates with numerous alterations in metabolic pathways, including those involved in glycolysis, ECM remodeling, and oxidative stress. Notably, the levels of oxidative stress markers, particularly 4-HNE, are markedly elevated in tear samples (Kannan et al., 2025). These observations provide novel insights for the formulation of personalized therapeutic strategies designed to modulate autophagy. These strategies may involve the restoration of corneal homeostasis through the activation of autophagic pathways or the integration of antioxidant treatments.

FECD is a progressive degenerative disorder affecting the corneal endothelium, and its progression is influenced by both genetic predispositions and advancing age. This condition is characterized by the thickening of Descemet’s membrane, a decrease in the population of endothelial cells, and the accumulation of extracellular matrix components, ultimately leading to a decrease in visual acuity. Recent research has revealed that the pathophysiology of FECD is significantly associated with mitochondrial dysfunction and ER stress. These factors can modulate abnormal mitophagy by targeting of the PINK1-Parkin signalling pathway, inhibiting *ATF4* to ameliorate disruptions in ER-mitochondrial interactions, activate the P62-autophagy-Keap1/Nrf2 pathway to bolster antioxidant defences or modulate autophagy-related genes, such as *DRAM1*, which may contribute to the deceleration of disease progression (Kumar and Jurkunas, 2021).

Research focused on therapeutic interventions for HSK has underscored the critical significance of autophagy modulation in the context of antiviral treatments. Investigations have shown that the infection caused by HSV1 increases its replication by activating the ATM signalling pathway, concurrently hindering the autophagic processes of the host. Conversely, the activation of autophagy, for instance, through the use of MG132, has been found to substantially suppresses viral replication, highlighting the potential of autophagy modulation as a therapeutic strategy for HSV1 infections (Yakoub and Shukla, 2015). Autophagy can inhibit autophagy by promoting ULK1 phosphorylation by the JAG1/NOTCH1 signalling pathway, and its inhibitor DAPT can relieve autophagy inhibition to reduce damage or increase the activity of the autophagy receptor OPTN to degrade viral proteins through selective autophagy; moreover, regulating dendritic cells to restore autophagy can reduce CD4+ T-cell-mediated inflammation. As an immune-mediated chronic inflammatory disease, autophagy presents a “bidirectional” role in HSK pathogenesis and provides multiple prospects for treatment (Chang et al., 2024).

Moderate enhancement of autophagy can mitigate inflammation on the ocular surface, alleviate oxidative stress, and enhance tear production and corneal health by utilizing autophagy activators such as rapamycin and trehalose (Hernandez et al., 2021). Targeting pathways associated with mitophagy, including AMPK/MFF and BNIP3L/NIX/MFN2, facilitates the clearance of impaired mitochondria, thus preserving metabolic equilibrium at the ocular surface (Stuard Sambhariya et al., 2023). Additionally, rectifying autophagy deficiencies, for instance, by modulating *DITT4* and stimulating the VDR pathway, can counteract cellular damage (Wang et al., 2019). These strategies underscore the therapeutic potential of autophagy modulation in maintaining ocular surface integrity and function.

Autophagy exhibits a dualistic role across various diseases, stages, and cell types, necessitating a tailored approach to its regulation in each scenario to prevent a “one-size-fits-all” treatment strategy (Xue et al., 2024). Furthermore, current autophagy modulators like rapamycin and chloroquine lack specificity and can induce systemic adverse effects, such as immunosuppression or lysosomal dysfunction (Xue et al., 2023). There is an imperative need to develop targeted ocular delivery systems, including nanoparticles and sustained-release hydrogels, to enhance local drug concentrations and minimize off-target impacts.

Additionally, while gene therapies, such as AAV-mediated gene delivery or the correction of mutations, show promise, the efficacy and long-term safety of vector penetration through the blood-retinal barrier must be thoroughly evaluated (Chalasani et al., 2014). Finally, the absence of precise biomarkers hampers the execution of personalized medicine. Future research should integrate single-cell sequencing with liquid biopsy techniques to dynamically monitor the relationship between autophagic flux, as indicated by markers like the LC3-II/p62 ratio, and disease progression. This integrated approach will facilitate the development of more nuanced and effective therapeutic interventions for autophagy-related ocular diseases.

## 5 Conclusion

Autophagy, as a central mechanism for preserving ocular tissue homeostasis, is involved in a multitude of pathological processes affecting the eye, from the cornea to the retina. It holds multidimensional promise as a therapeutic avenue for various eye conditions (Singh and Devi, 2021). By focusing on the pathways autophagy regulatory, promoting selective autophagy such as mitophagy, and manipulating related molecules, such as TFEB, innovative treatment strategies for eye diseases are anticipated to emerge. These approaches are particularly promising because of their potential to slow the degeneration of retinal cells, safeguard corneal function, and preserve the transparency of the lens.

However, the dual role of autophagy, challenges in ocular tissue-specific delivery, the complexity of disease mechanisms, and safety issues with clinical translation are still key challenges that need to be overcome (Gong et al., 2021). Based on current research advancements, key directions for advancing precise and controllable clinical translation of autophagy-targeted therapies include developing targeting peptide-modified nanocarriers, exosomes, or viral vectors to enable targeted drug delivery to diseased cells; selectively regulating key autophagy nodes according to specific ocular diseases; utilizing autophagy-related biomarkers such as LC3-II and p62 for dynamic monitoring to guide individualized treatment; and conducting preclinical validation of the efficacy and safety of treatments using patient-derived iPSC models and gene-edited animal platforms.

The clinical translation of autophagy-targeted therapies for ocular diseases faces three core challenges. First, the majority of findings are derived from animal models and *in vitro* cellular experiments and have yet to be translated into clinically applicable treatments. Rigorous evaluations of therapeutic efficacy in human subjects are lacking, and the complex heterogeneity of human diseases—along with varying responses across different disease stages—makes determining the optimal treatment window and validating its effectiveness difficult.

In addition, safety data are scarge. While certain autophagy modulators, such as chloroquine have shown potential in preclinical models, their long-term systemic or local application may disrupt cellular homeostasis, induce retinal toxicity, or cause systemic off-target effects. These risks have not yet been systematically assessed through well-controlled clinical trials.

Finally, the unique anatomical structure and physiological barriers of the eye present significant challenges for drug delivery. Barriers such as the blood retinal barrier and the corneal barrier limit the effective accumulation of systemically administered drugs in ocular tissues. Although researchers have explored various delivery strategies, including hydrogel-based sustained-release systems and nanocarriers, achieving targeted and efficient delivery while preserving drug activity remains a pressing technical hurdle. These issues are especially critical for chronic ocular conditions requiring long-term treatment, where the development of delivery systems capable of providing sustained and stable drug release is essential. Future research should prioritize the translation of these mechanistic insights, validation of safety and efficacy in human subjects, and ultimately bridge the gap between foundational research and clinical practice.

Future research needs to focus on a fine analysis of disease-specific autophagy regulatory networks, preclinical validation of highly selective small molecules or gene editing tools and the construction of multiomics-driven precision medicine models to verify the effectiveness of individualized intervention programs, and ultimately promote autophagy regulatory therapy from basic research to clinical application to provide a new theoretical basis and practical direction for eye disease treatment.
